# Cardiomyocyte *Oga* haploinsufficiency increases O-GlcNAcylation but hastens ventricular dysfunction following myocardial infarction

**DOI:** 10.1371/journal.pone.0242250

**Published:** 2020-11-30

**Authors:** Sujith Dassanayaka, Kenneth R. Brittian, Bethany W. Long, Lauren A. Higgins, James A. Bradley, Timothy N. Audam, Andrea Jurkovic, Anna M. Gumpert, Linda T. Harrison, István Hartyánszky, Péter Perge, Béla Merkely, Tamás Radovits, John A. Hanover, Steven P. Jones

**Affiliations:** 1 Department of Medicine, University of Louisville, Louisville, KY, United states of America; 2 Heart and Vascular Center, Semmelweis University, Budapest, Hungary, United states of America; 3 Laboratory of Cell and Molecular Biology, NIH-NIDDK, Bethesda, MD, United states of America; Scuola Superiore Sant'Anna, ITALY

## Abstract

**Rationale:**

The beta-O-linkage of N-acetylglucosamine (i.e., O-GlcNAc) to proteins is a pro-adaptive response to cellular insults. To this end, increased protein O-GlcNAcylation improves short-term survival of cardiomyocytes subjected to acute injury. This observation has been repeated by multiple groups and in multiple models; however, whether increased protein O-GlcNAcylation plays a beneficial role in more chronic settings remains an open question.

**Objective:**

Here, we queried whether increasing levels of cardiac protein O-GlcNAcylation would be beneficial during infarct-induced heart failure.

**Methods and results:**

To achieve increased protein O-GlcNAcylation, we targeted *Oga*, the gene responsible for removing O-GlcNAc from proteins. Here, we generated mice with cardiomyocyte-restricted, tamoxifen-inducible haploinsufficient *Oga* gene. In the absence of infarction, we observed a slight reduction in ejection fraction in *Oga* deficient mice. Overall, *Oga* reduction had no major impact on ventricular function. In additional cohorts, mice of both sexes and both genotypes were subjected to infarct-induced heart failure and followed for up to four weeks, during which time cardiac function was assessed via echocardiography. Contrary to our prediction, the *Oga* deficient mice exhibited exacerbated—not improved—cardiac function at one week following infarction. When the observation was extended to 4 wk post-MI, this acute exacerbation was lost.

**Conclusions:**

The present findings, coupled with our previous work, suggest that altering the ability of cardiomyocytes to either add or remove O-GlcNAc modifications to proteins exacerbates early infarct-induced heart failure. We speculate that more nuanced approaches to regulating O-GlcNAcylation are needed to understand its role—and, in particular, the possibility of cycling, in the pathophysiology of the failing heart.

## Introduction

Numerous post-translational modifications (PTMs) regulate protein function, and by extension, cellular function. One such PTM, the beta-O-linkage of N-acetylglucosamine (i.e., O-GlcNAc) to serine/threonine residues of proteins has emerged as an important post-translational modification of a number of nucleocytoplasmic proteins. There are two mammalian genes validated as regulators of the O-GlcNAc modification on proteins. These genes are O-GlcNAc transferase (*Ogt*) and O-GlcNAcase (*Oga* in mice; *OGA* in humans), and the proteins they encode are OGT and OGA, respectively. OGT adds the O-GlcNAc modification to serine/threonine residues and OGA is the only known enzyme that removes the O-GlcNAc modification. Changes in protein O-GlcNAcylation have been implicated in a variety of pathophysiological processes, including diseases of the cardiovascular system [1–6].

Zachara et al [7] first suggested that protein O-GlcNAcylation may function a stress signal. Subsequent studies from our group[3, 8–12] and others[2, 13–18] indicated that O-GlcNAcylation was indeed a beneficial stress signal in the context of acute cardiomyocyte damage. Although we[3, 8, 9, 11, 12] and others[2, 13–18] provided evidence that O-GlcNAc protects cardiomyocyte survival (in part by favorably affecting mitochondrial function), the complete picture of the cytoprotective mechanisms of O-GlcNAc remains unfinished. Efforts to identify O-GlcNAc modified proteins could aid progress in this area; however, there are other, perhaps more significant and proximal, issues that we addressed in the present study.

Despite a relative consensus of the aforementioned beneficial view of O-GlcNAc in acute injury of the heart, the role of O-GlcNAc in chronic conditions is less clear [19, 20]. Several studies implicate O-GlcNAc in the pathogenesis of diabetes [21–25]; however, an absolute consensus has not yet emerged, and seemingly conflicting reports exist [26–29]. Thus, the role of O-GlcNAcylation in chronic conditions must be resolved. To this end, we have previously investigated the role of OGT in a murine model of infarct-induced heart failure [19]. There, we found that the induction of OGT was likely a pro-adaptive response in the heart to attenuate the severity of heart failure. On face value, such results suggest that, at least in the heart, the role of OGT (and perhaps enhanced O-GlcNAcylation) performs beneficial functions. In other words, suppression of OGT (and, by extension, decreasing O-GlcNAcylation) exacerbates heart failure. Conversely, suppression of OGA (and, by extension, increasing O-GlcNAcylation) might attenuate heart failure. Thus, we tested the hypothesis that reduction of OGA (*Oga*) augments O-GlcNAc levels and attenuates the severity of heart failure. The results of this study are important for generating insights regarding O-GlcNAcylation in chronic conditions, but most directly for understanding the role of O-GlcNAcylation in the failing heart.

## Methods

All animal procedures were performed in accordance with the National Institutes of Health Guide for the Care and Use of Laboratory Animals and were approved by the University of Louisville Institutional Animal Care and Use Committee.

### Generation of inducible, cardiac-specific *Oga* deficient mice

We received heterozygous *Oga* loxP-flanked (i.e., *Oga*^*fl/+*^*)* mice from the Hanover lab [30]. Progeny from these mice were then bred with *Myh6*-driven MerCreMer (aka, MCM) transgenic mice (A1cf^Tg(Myh6-cre/Esr1*)1Jmk^/J, Jackson Laboratory 005650) to generate tamoxifen-inducible, cardiomyocyte-specific, heterozygous deletion of *Oga* (icm*Oga*^*+/-*^), as illustrated in S1 Fig. We did not use homozygous floxed (i.e., *Oga*^*fl/fl*^) mice, in line with previous recommendations [30]. These animals are perinatal lethal and physiological studies have relied upon induced haploinsufficiency [30]. Instead, we used MCM^+/-^; *Oga*^fl/+^ mice as our OGA deficient mice and MCM^-/-^; *Oga* MCM^+/-^ mice as the control. In this paper, we refer to these mice as icm*Oga*^*+/-*^ and icm*Oga*^*+/+*^, respectively. Both groups received tamoxifen, as detailed below. All mice used in this study were on a C57BL/6J background.

### Genotyping of transgenic mice

At 3–4 weeks of age, mice were ear tagged, and tail snips were taken. Total DNA was isolated from tail snips using the Qiagen DNeasy Tissue Kit. The DNA was stored at -20°C until PCR was performed. Genotyping PCR primers used for this study are listed in S1 Table. PCR was performed using the Taq PCR Core Kit from Qiagen. Mixes were created as follows: tube 1 contained 1 μl DNTP, 1 μL of 20 μmol/L forward primer 1 μL of 20 μmol/L reverse primer, 10 μL Enzyme Q, and 7 μL water per sample. Tube 2 contained 5 μL 10× buffer, 0.5 μL Taq, and 14.5 μL water per sample. 20 μL of each tube were added to a PCR tube containing 10 μL of purified DNA. PCR was performed at the following conditions: 1 cycle of 94°C for 3 min, 35 cycles of 94°C for 30 sec, 61°C for 1 min and 72°C for 1 min, 1 cycle of 72°C for 2 min then held at 4°C *ad infinitum*. PCR samples were then run on a 1.2% agarose gel for MCM and 2% agarose gel for *Oga* flox with SYBR Safe stain (Invitrogen). Gels were visualized under UV light using a Fuji LAS-3000 imaging system.

### Tamoxifen treatment

Recombination was induced with tamoxifen to generate icm*Oga*^*-/-*^ mice. Tamoxifen was prepared by dissolving 4-hydroxytamoxifen (25 mg, Sigma, St. Louis, MO) in 1 mL of warmed (37°C) 100% ethanol. The mixture was vortexed and sonicated until fully dissolved. Then the mixture was added to 9 mL peanut oil (Sigma, St. Louis, MO) and was vortexed and sonicated until suspended. A bolus of 4-hydroxytamoxifen (20 mg/kg) was injected intraperitoneally on alternating sides of icm*Oga*^*+/-*^ *and* icm*Oga*^*+/+*^ littermates aged 10–18 wk old consecutively for 5 d. Residual 4-hydroxytamoxifen was allowed to “wash out” for 5 d prior to experimentation. Mice were subjected to echocardiography 10 d following the initial injection of tamoxifen and at 1 or 4 wk post-MI. Mice were subjected to MI 1 wk after, at age 12–20 wk. See S1C Fig for timeline of experimental procedures.

### Echocardiography

Naïve icm*Oga*^*+/-*^ *and* icm*Oga*^*+/+*^ littermates (both sexes) were subjected to baseline and 8 wk post-tamoxifen echocardiography. Icm*Oga*^*+/-*^ *and* icm*Oga*^*+/+*^ littermates designated for MI were subjected to 1 and 4 wk post-MI echocardiography. Transthoracic echocardiography of the left ventricle was performed as described previously [19, 31, 32]. The sonographer was blinded to mouse genotype. Transthoracic echocardiography of the left ventricle was performed with a Vevo 770 echocardiography system. Mice body temperature was maintained at 36.5–37.5°C using a rectal thermometer interfaced with a servo-controlled heat lamp. Mice were anesthetized with 2% isoflurane then maintained under anesthesia with ~1.5% isoflurane. Using the Vevo rail system, the mouse was placed supine on an examination board interfaced with the Vevo 770. Next, depilatory cream was applied to the mouse’s chest and wiped clean to remove fur. The 707-B (30 MHz) scan head was used to obtain 2D images (100 fps) of the parasternal long axis; M-mode images were also acquired from this position. The probe was then rotated 90° to acquire short axis views. Beginning at the base and moving apically, serial 2D images were taken every millimeter. All measurements were taken with the aid of the Vevo 770’s rail system to maintain probe placement and allow for precise, minute adjustments of probe position along the long axis. Left ventricular diameters during diastole and systole (LVIDd and LVIDs) were determined from long axis M-modes along with heart rate (HR). Left ventricular fractional shortening (%FS) was calculated as: ((LVIDd- LVIDs)/LVIDd) × 100%. Diastolic and systolic volumes were determined by applying Simpson’s rule of discs to the serially acquired short axis images. Stroke volume (SV) was calculated as: diastolic volume—systolic volume. Ejection Fraction was calculated as: (SV/Diastolic Volume)*100%. Cardiac output was determined by: SV × HR.

### Myocardial infarction

icm*Oga*^*+/-*^ and icm*Oga*^*+/+*^ littermates aged 12–20 wk old mice (both sexes) were subjected to non-reperfused myocardial infarction (MI) as described previously [19, 31, 32]. Briefly, mice were anesthetized with intraperitoneal injections of ketamine hydrochloride (50 mg/kg) and sodium pentobarbital (50 mg/kg). Mice were orally intubated and ventilated; the ventilator’s room-air port was supplemented with oxygen. A 7–0 silk suture was passed under the left coronary artery and tied. The chest and skin were closed. Mice were extubated upon recovery of spontaneous breathing. Analgesia (ketoprofen, 5 mg/kg) was provided prior to recovery and by 24 and 48 h post-surgery. The surgeon was blinded to mouse genotype. Seven days after MI, all mice were subjected to an echocardiogram to confirm sufficient depression of cardiac function (LVEF<60%). Mice were followed up to 4 wk. Any mouse with LVEF>60% was excluded from the study.

### Reverse transcriptase PCR and real-time PCR

The total RNA from the LV was extracted and used to make cDNA as described previously [19, 31, 33]. The relative levels of mRNA transcripts were quantified by real-time PCR using Power SYBR Green (Thermo Fisher Scientific) on a real-time PCR system (ABI 7900 HT, Applied Biosciences). Most primers were made using NCBI Primer Blast except HPRT primers (PPM03559E-200, QIAGEN). The data were normalized to mouse HPRT mRNA threshold cycle (C_T_) values by using the ΔΔC_T_ comparative method. Primer sequences are listed in S2 Table.

### Protein isolation

Protein was harvested from cardiac tissue as described previously [19, 33]. Protein concentrations were determined by the Bradford assay with Bio-Rad protein assay dye reagent (Bio-Rad Laboratories) and using different concentrations of bovine serum albumin as standards. Protein concentrations were measured with a Thermo Electron Type 1500 Multiskan Spectrum Microplate Reader and SkanIt RE for MSS 2.2 software.

### Immunoblotting

Protein samples were subjected to electrophoresis in SDS-PAGE gels (4–12%, Invitrogen) and transferred to PVDF membranes (Immobilon-P, EMD Millipore) at 4°C. For O-GlcNAc immunoblotting, membranes were allowed to dry at room temperature for 1 h. The blot was then probed with primary antibody against O-GlcNAc (clone: RL2; 1:1000, Affinity Bioreagents) in PBS-casein (Bio-Rad Laboratories) overnight at 4°C. Membranes were washed three times with 1x PBS. Membranes were incubated at room temperature with secondary antibody (goat anti-mouse IgG-HRP; 1:4000, sc-2005; Santa Cruz Biotechnology) in PBS-casein. Membranes were again washed three times with 1× PBS and then imaged. All other western blotting followed standard protocols. Briefly, membranes were blocked at room temperature using Tris-buffered saline pH 7.5 (TBS) containing nonfat milk (5%), washed with TBS containing Tween-20 (TBS-T, 0.1%), and probed with primary antibody. Antibodies for OGT (D1D8Q—1:2000, Cell Signaling), OGA (NCOAT—1:1000, Santa Cruz Biotechnology), and α-tubulin (T6074–1:2000, Sigma-Aldrich) were made in TBS containing nonfat milk (1%). After overnight incubation at 4°C, blots were washed in TBS containing Tween-20 (TBS-T, 0.1%). The blots were blocked for 15 min in TBS-T containing 1% milk, washed, and then incubated with goat anti-rabbit IgG-HRP (sc-2004; Santa Cruz Biotechnology or 7074; Cell Signaling Technology) or goat anti-mouse IgG-HRP (Santa Cruz Biotechnology), in 1:2000 dilution (for OGT, OGA, and α-tubulin). After washing three times with TBS-T, the membrane was saturated with SuperSignal West Pico Chemiluminescent Substrate (Thermo Fisher Scientific) and imaged on a Fuji LAS-3000 bio-imaging analyzer. To confirm the linear range of the signal, multiple exposures from every experiment were performed. Each lane was normalized to a control protein (α-tubulin) or total protein content (via Ponceau stain) and expressed as relative to control (set as 100%).

### Pathology

Following final echocardiography, hearts were excised, manually perfused to remove most of the blood, and arrested in diastole with 60 mM KCl in 1× PBS. Hearts were then sectioned into 1 mm short-axis sections. A mid-ventricular section from each heart was fixed with 10% neutral-buffered formalin for 24 h and stored in 70% ethanol until tissue processing. The samples were later embedded, cut, and mounted. Later, the slides were deparaffinized and rehydrated as needed for the appropriate stain. For all analyses described below, the microscopist was blinded to group assignment.

#### Cardiomyocyte hypertrophy

Cardiac sections were stained with 5 μg/mL of wheat germ agglutinin (WGA; AlexaFluor 555 conjugate; Invitrogen) to identify cell borders and stained with 1 mg/mL of DAPI to detect nuclei. WGA-stained cells were visualized using a Nikon TE-2000E2 microscope interfaced with a Nikon A1 confocal system. A 405 nm laser was used to excite DAPI and 450/50 emission filter was used; 561 nm laser was used to excite TRITC (i.e., WGA label) and a 595/50 emission filter was used. Cell areas were measured using Nikon Elements software [64-bit version 3.22.00(Build 710)]. Cardiomyocytes where chosen based on their circularity and whether they had centrally located nuclei. Circularity was calculated using the Shape Factor feature in NIS-Elements AR 4.0. Cardiomyocytes were chosen based on a Shape Factor between 1.0 and 0.895 (radius ratio of 1:1 to 1:1.4).

#### Capillary density

Cardiac sections were stained with 40 μg/mL isolectin B4 (Fluorescein labeled *Griffonia simplicifolia* Lectin I; Vector Labs) and imaged as described previously [32]. Capillary density was determined by dividing the total number of isolectin B4 positive vessels by the area of the image (number of capillaries per mm^2^).

#### Cardiac apoptosis

A TUNEL assay kit (TB235, Promega Corporation) was used on LV sections according to the manufacturer’s instructions. Sections were also stained with DAPI to identify nuclei. Sections were imaged using an epi-fluorescence microscope (Nikon Eclipse Ti) using a 20x objective. TUNEL positivity was calculated by dividing the total number of TUNEL positive cells by the number of nuclei.

### Human heart samples

Well-characterized de-identified human myocardial tissue samples were obtained from the Transplantation Biobank of the Heart and Vascular Center at Semmelweis University, Budapest, Hungary [34, 35]. Following institutional and national ethical committee approval (ethical permission numbers: ETT TUKEB 7891/2012/EKU (119/PI/12.) and TUKEB 73/2005.) and informed consent from patients, myocardial tissue samples were surgically removed, immediately frozen in liquid nitrogen, and stored at −80°C. Human heart samples were collected from males and females (see S6 Table for characteristics). Control samples were isolated from papillary muscle biopsies in patients undergoing mitral valve replacement surgery. Failing heart samples were biopsied from the anterior wall of the left ventricle in end-stage heart failure patients undergoing heart transplantation. Echocardiography data were obtained from the database of the Transplantation Biobank.

### Statistical analysis

Results are shown as mean or mean ± SD. Statistical analysis (GraphPad 8.0.2(159)) was conducted using a two-tailed Student’s *t* test, when appropriate. A chi-squared test was used to determine statistical differences in patient demographics. Differences were considered statistically significant if *p*<0.05.

## Results

### icm*Oga*^*+/-*^ mice exhibit normal cardiac function and augmented protein O-GlcNAcylation

**Fig 1:**
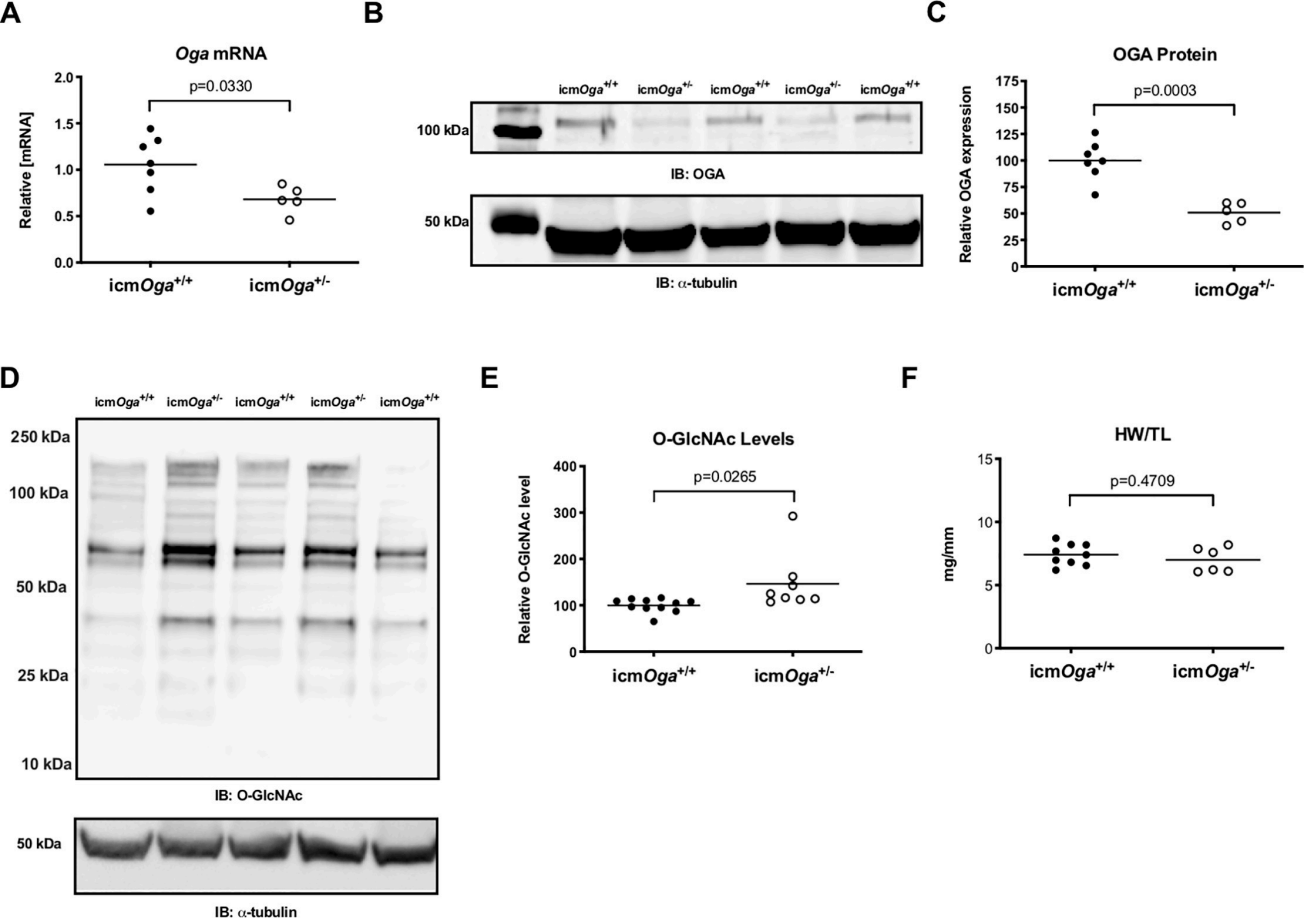
*Oga* deficiency induces elevated cardiac protein O-GlcNAcylation. Cardiac tissue from naïve male icm*Oga*^+/+^ (n = 5) and icm*Oga*^+/-^ (n = 7) mice was harvested at 12–20 wk of age. Cardiac *Oga* mRNA expression (A). Immunoblot (B) and densitometric analysis (C) of OGA protein expression. Western blot for protein O-GlcNAcylation in icm*Oga*^+/+^and icm*Oga*^+/-^ hearts (D) and densitometric analysis (E). Gravimetric analysis of heart weight to tibia length (F). An unpaired Student’s *t*-test was used to determine significance between icm*Oga*^+/+^ and icm*Oga*^+/-^ groups.

Dysregulation of O-GlcNAc metabolizing enzymes is known to be associated with heart failure [19, 31, 33, 36, 37]. Previously, we ablated cardiac *Ogt* [19]. Here, we designed a study to determine the role of OGA after MI. In order to do so, we bred and characterized a cardiomyocyte-specific, inducible *Oga* deficient mice. Mice with one *Oga* floxed allele were crossed with α-MHC MCM mice to generate icm*Oga*^+/-^ and icm*Oga*^+/+^ littermates.(S1A and S1B Fig). Zygotically inherited heterozygous animals were previously used to examine the metabolic consequences of OGA insufficiency and are known to exhibit metabolic and transcriptional defects due to elevated O-GlcNAc levels [30]. The current study employs the same strategy but conditionally targeted to cardiomyocytes. Naïve icm*Oga*^*+/-*^ and icm*Oga*^*+/+*^ littermates were subjected to tamoxifen treatment and hearts were harvested 5 d post washout. As expected, *Oga* mRNA and protein expression was markedly reduced in icm*Oga*^*+/-*^ hearts (Fig 1A–1C). Moreover, we showed that this reduction in OGA was limited to the heart (S2A–S2C Fig). Protein O-GlcNAcylation was significantly augmented in icm*Oga*^*+/-*^ hearts (Fig 1D and 1E). Heart weights were similar between icm*Oga*^*+/+*^ and icm*Oga*^*+/-*^ groups (Fig 1F). A separate set of naïve icm*Oga*^*+/-*^ and icm*Oga*^*+/+*^ littermates were subjected to echocardiography 8 wk post-tamoxifen treatment to establish whether cardiac *Oga* deletion *per se* affects cardiac function. Ejection fraction was slightly reduced in surgically naïve icm*Oga*^*+/-*^ (S3 Table), though no other end points differed. Thus, reduction of cardiomyocyte *Oga* in surgically naïve mice did not produce a significant cardiac phenotype but did augment cardiac protein O-GlcNAcylation. Given this observation, we next determined whether insufficiency of cardiomyocyte *Oga* attenuates infarct-induced cardiac dysfunction, which is the central focus of this study.

### Targeting of cardiomyocyte Oga exacerbates cardiac dysfunction early after myocardial infarction in male mice

**Fig 2:**
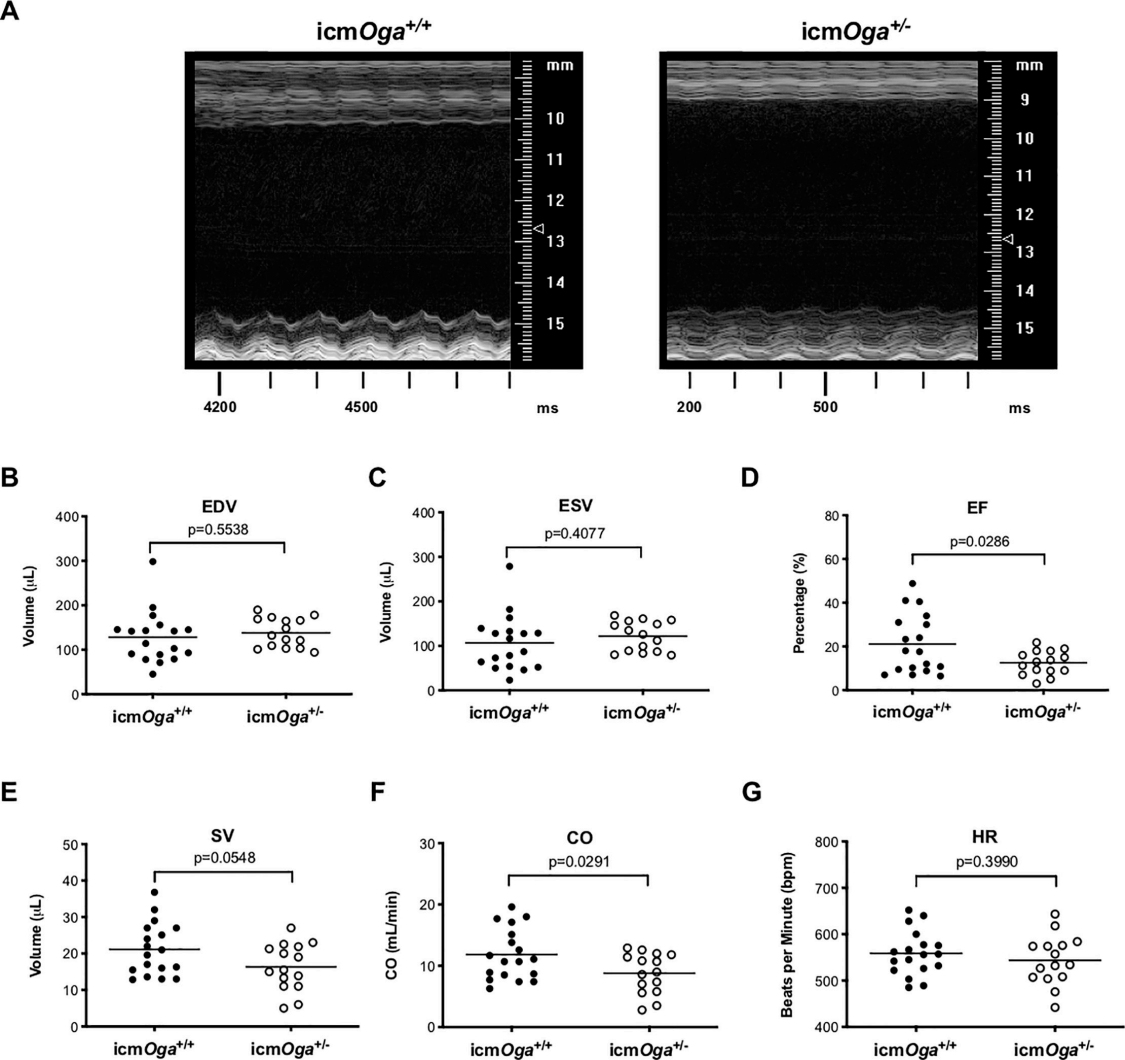
*Oga* deficiency exacerbates cardiac dysfunction 1 wk post-MI. Male, tamoxifen treated icm*Oga*^+/+^ (n = 18) and icm*Oga*^+/-^ (n = 15) were subjected to echocardiography after 1 wk post-MI. Representative m-mode images of icm*Oga*^+/+^and icm*Oga*^+/-^ hearts (A). Left ventricular end diastolic volume; EDV (B). Left ventricular end systolic volume; ESV (C). Left ventricular ejection fraction; EF (D). Left ventricular stroke volume; SV (E). Heart rate; HR (F). Cardiac output; CO (G). An unpaired Student’s *t*-test was used to determine significance between icm*Oga*^+/+^ and icm*Oga*^*+/-*^ groups.

To determine the role of cardiomyocyte *Oga* deficiency during HF, tamoxifen treated male icm*Oga*^*+/+*^ and icm*Oga*^*+/-*^ mice were subjected to MI at 12–20 wk old and followed for 1 wk (see timeline in S1C Fig). Cardiac function was assessed by echocardiography 1 wk post-MI (Fig 2 and Table 1). M-mode echocardiograms of icm*Oga*^*+/+*^ and icm*Oga*^*+/-*^ hearts were acquired. Representative pictures are shown in Fig 2A. Interestingly, icm*Oga*^*+/-*^ mice exhibited more ventricular dysfunction. In icm*Oga*^*+/-*^ hearts, both LV diastolic (Fig 2B) and systolic volumes (Fig 2C) were unchanged. Ejection fraction (Fig 2D) was significantly decreased in icm*Oga*^*+/-*^ mice. Stroke volume and heart rate were not different (Fig 2E and 2F). Cardiac output was significantly decreased (Fig 2G). No changes were observed in diastolic and systolic inner ventricular diameters, fractional shortening, and diastolic and systolic posterior or anterior wall thicknesses (Table 1). Female icm*Oga*^*+/+*^ and icm*Oga*^*+/-*^ mice subjected to 1 wk MI display no differences in cardiac function (S4 Table). Contrary to our central hypothesis, these data indicate that reduction of cardiomyocyte *Oga* exacerbates infarct -induced cardiac dysfunction.

**Table 1:** Echocardiography data at 1 wk post-MI.

	icm*Oga*^*+/+*^	icm*Oga*^*+/-*^	p value
**BW (g)**	27 ± 2	27 ± 4	0.3633
**LVIDd (mm)**	5.8 ± 1.2	6.1 ± 0.8	0.3314
**LVIDs (mm)**	5.2 ± 1.4	5.7 ± 0.9	0.2172
**FS (%)**	12 ± 8	7 ± 4	0.0599
**LVPWd (mm)**	0.6 ± 0.4	0.5 ± 0.4	0.5127
**LVPWs (mm)**	0.8 ± 0.5	0.7 ± 0.5	0.4463
**LVAWd (mm)**	0.4 ± 0.3	0.4 ± 0.1	0.6589
**LVAWs (mm)**	0.5 ± 0.4	0.4 ± 0.2	0.3963

Tamoxifen treated male icm*Oga*^+/+^ (n = 18) and icm*Oga*^+/-^ (n = 15) were subjected to echocardiography after 1 wk post-MI. Indices of left ventricular cardiac function were assessed. No significant changes were observed in left ventricular inner systolic diameter (LVIDs), left ventricular inner diastolic diameter (LVIDd), fractional shortening (FS), left ventricular posterior wall thickness in diastole (LVPWd), left ventricular posterior wall thickness in systole (LVPWs), and left ventricular anterior wall thickness in diastole or systole (LVAWd, LVAWs). An unpaired Student’s *t*-test was used to determine significance between icm*Oga*^+/+^ and icm*Oga*^*+/-*^ groups.

### icm*Oga*^*+/-*^ diminished cardiac OGA without affecting protein O-GlcNAcylation early after MI

**Fig 3:**
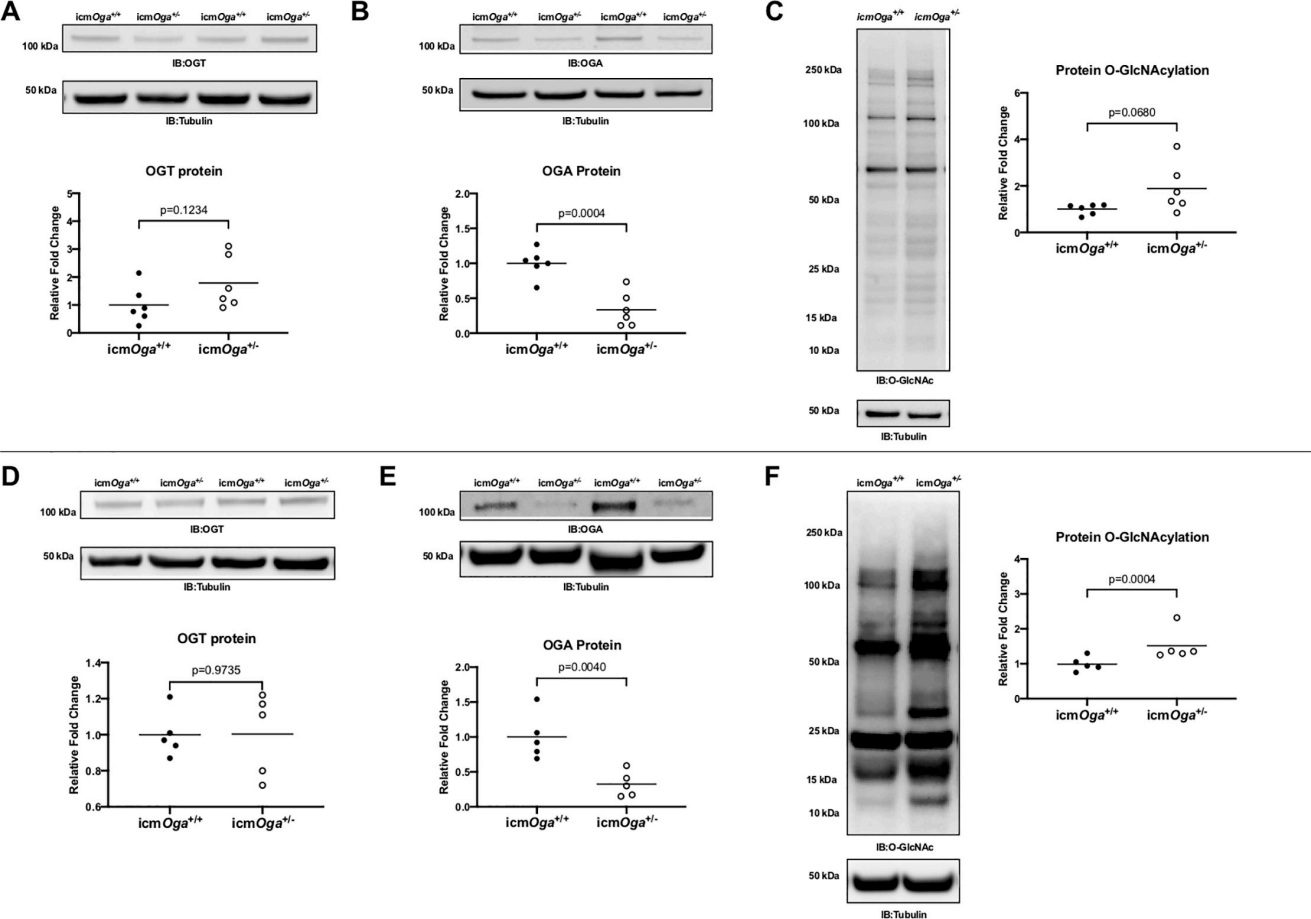
Deficiency of *Oga* diminishes cardiac OGA. Cardiac tissue harvested from 1 or 4 wk post-MI icm*Oga*^+/+^ and icm*Oga*^*+/-*^ hearts were probed for OGT, OGA, and protein O-GlcNAcylation. Immunoblot and densitometric analysis for OGT (A), OGA (B), and protein O-GlcNAcylation (C) at 1 wk post-MI. Immunoblot and densitometric analysis for OGT (D), OGA (E), and protein O-GlcNAcylation (F) at 4 wk post-MI. An unpaired Student’s *t*-test was used to determine significance between icm*Oga*^*+/-*^ and icm*Oga*^*+/+*^groups.

To determine whether *Oga* reduction altered regulation of O-GlcNAcylation, we queried whether OGA and OGT protein expression was different in icm*Oga*^*+/-*^ and icm*Oga*^*+/+*^ hearts 1 wk post-MI. We probed for OGT, OGA, and protein O-GlcNAcylation via immunoblot (Fig 3A–3C). OGA expression was significantly diminished (Fig 3B). No overall changes in OGT or protein O-GlcNAcylation were observed (Fig 3A–3C). *Oga* haploinsufficiency reduced OGA expression without significantly altering cardiac protein O-GlcNAcylation.

### Targeting of OGA did not affect cardiomyocyte hypertrophy, capillary density, or apoptosis

To determine how *Oga* deficiency contributed to the exacerbation of post-MI cardiac dysfunction at 1 wk post-MI, we measured cardiomyocyte hypertrophy, capillary density, and apoptosis. Molecular markers of hypertrophy (*Nppa* and *Nppb*) between icm*Oga*^*+/-*^ and icm*Oga*^*+/+*^ hearts were not significantly different (S3A Fig). As expected, heart weights were also similar (S3B Fig). To rule out whether *OGA* deficiency acutely affects cardiomyocyte size following MI, we measured cardiomyocyte cross-sectional area (S3C and S3D Fig). Cardiomyocyte cross-sectional area was not different in border, ischemic, or remote zones (S3D Fig). To account for the deterioration in function, we interrogated whether capillary density was different in mid-ventricular sections of the heart (S3C and S3E Fig). Capillary densities were not different between groups. To further characterize how icm*Oga*^*+/-*^ exacerbates cardiac dysfunction, we examined whether cardiac apoptosis was altered in between icm*Oga*^*+/-*^ and icm*Oga*^*+/+*^ hearts. *Bcl2*, a regulator of apoptosis, was not significantly different (S4A Fig). In addition, TUNEL staining revealed no significant changes in apoptosis (S4B Fig). Thus, targeting of *Oga* does not impact cardiomyocyte cross-sectional area, capillary density, or apoptosis at early time points following MI.

### Oga deficiency does not maintain exacerbation of cardiac dysfunction in chronic HF

Although the exacerbation in cardiac dysfunction at 1 wk was significant, we wanted to know whether such deterioration in function was maintained by extended observation (i.e., 4 wk post-MI). Infarcted icm*Oga*^+/+^ and icm*Oga*^+/-^ mice were subjected to echocardiography at 1 wk post-MI. This same subset of mice was profiled again at 4 wks. Icm*Oga* deletion did not affect cardiac function 4 wk post-MI (S5A Fig and S5 Table). Interestingly, the significant deterioration in cardiac function at 1 wk-post MI was limited to 1 wk time point. These data indicate that the exacerbated cardiac dysfunction exhibited by *Oga*-deficient mice at 1 wk post-MI was not maintained at 4 wk-post MI.

### Cardiac protein O-GlcNAcylation is upregulated in failing icm*Oga*^*+/-*^ hearts

To determine whether ablating *Oga* dysregulated protein O-GlcNAcylation, we queried whether OGT protein expression and overall protein O-GlcNAcylation was different in icm*Oga*^*+/-*^ and icm*Oga*^*+/+*^ hearts at 4 wk post-MI. Although OGT protein expression was also not different (Fig 3D), OGA protein expression was reduced in icm*Oga*^*+/-*^ hearts (Fig 3E). Protein O-GlcNAcylation was upregulated in icm*Oga*^*+/-*^ hearts (Fig 3F). O-GlcNAc levels were elevated in *Oga*-haplo-insufficient cardiomyocytes during chronic heart failure, suggesting that loss of regulation by OGA may affect O-GlcNAcylation in the failing heart.

### Reduction of *Oga* altered ischemic and remote cardiomyocyte size, but not capillary density, or apoptosis in chronic HF

**Fig 4:**
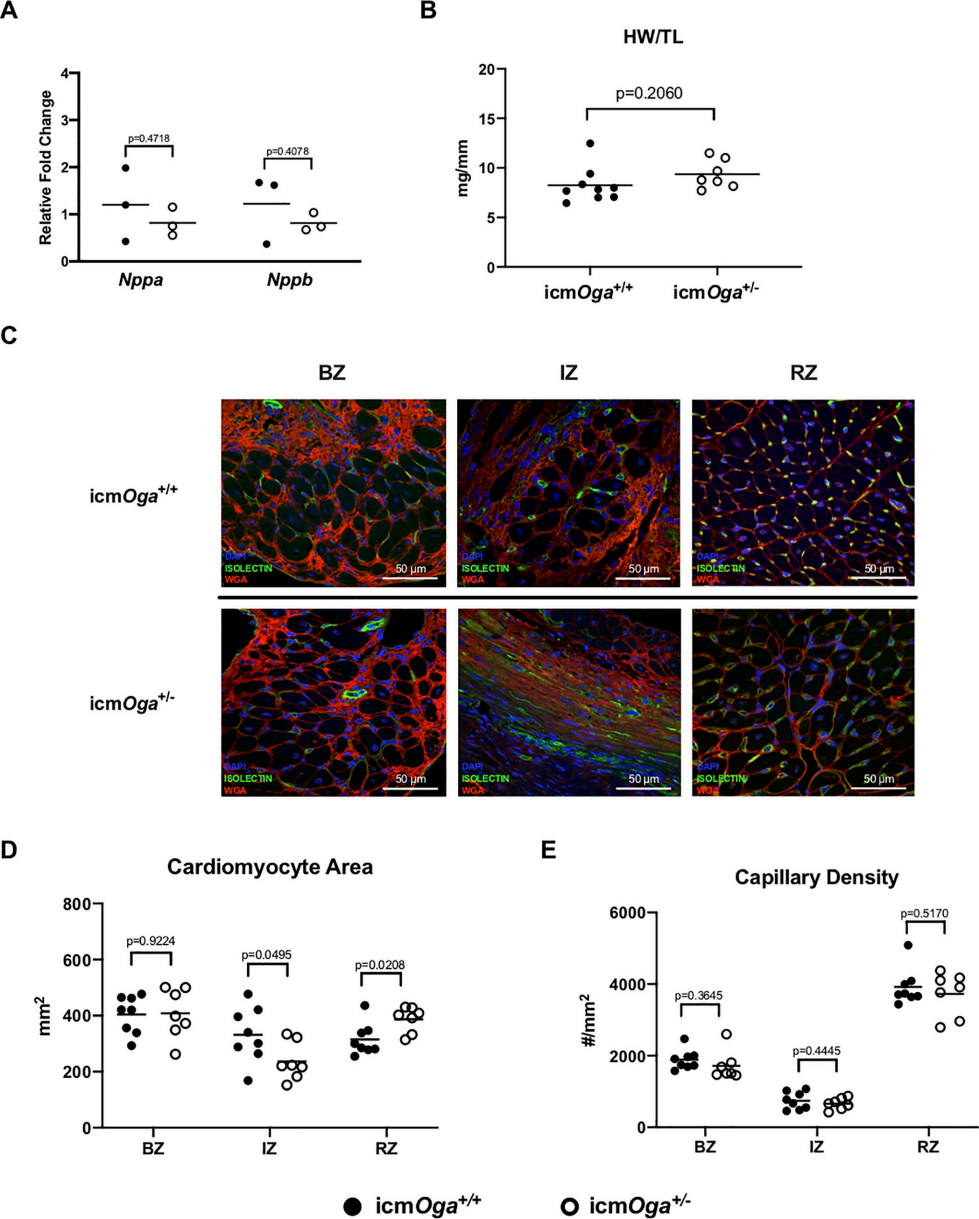
Cardiac *Oga* haploinsufficiency alters ischemic and remote cardiomyocyte size without affecting capillary density at 4 wk post-MI. Gene expression of markers of cardiac hypertrophy 4 wk post-MI (A). Gravimetric analysis of heart size; heart weight to tibia length (B). Representative images of WGA and isolectin-stained icm*Oga*^*+/-*^ and icm*Oga*^*+/+*^ heart sections (C). Cardiomyocyte cross-sectional area was measured in the area bordering the infarct (BZ), in the infarct zone (IZ), and remote (RZ) from the infarct (D). Capillary density in border (BZ), infarct (IZ), and remote zones (RZ) (E). An unpaired Student’s *t*-test was used to determine significance between icm*Oga*^*+/-*^ and icm*Oga*^*+/+*^groups.

To determine whether *Oga* deficiency contributed cardiac remodeling at 4 wk post-MI, we measured cardiomyocyte hypertrophy, capillary density, and apoptosis. As expected, molecular markers of hypertrophy (*Nppa* and *Nppb*) were also similar (Fig 4A). Heart weights between icm*Oga*^*+/-*^ and icm*Oga*^*+/+*^ hearts were not significantly different (Fig 4B).To rule out whether *OGA* deficiency affects cardiomyocyte size in chronic HF, we measured cardiomyocyte cross-sectional area (Fig 4C and 4D). We observed no changes in cardiomyocyte cross-sectional area in border zone. Interestingly, icm*Oga*^*+/-*^ cardiomyocytes were smaller in the ischemic zone and larger than icm*Oga*^*+/+*^ cardiomyocytes in the remote zones (Fig 4C and 4D). Furthermore, we interrogated whether capillary density was different in mid-ventricular sections of the heart (Fig 4E). Capillary densities were not different between groups. Finally, we examined whether cardiac apoptosis was altered in between icm*Oga*^*+/-*^ and icm*Oga*^*+/+*^ hearts. *Bcl2* expression was not significantly different (S6A Fig). Moreover, TUNEL staining revealed no significant changes in apoptosis (S6B and S6C Fig). Thus, perturbation of *Oga* levels hastens cardiac remodeling.

### Expression of O-GlcNAc metabolizing enzymes is augmented in human heart failure

**Fig 5:**
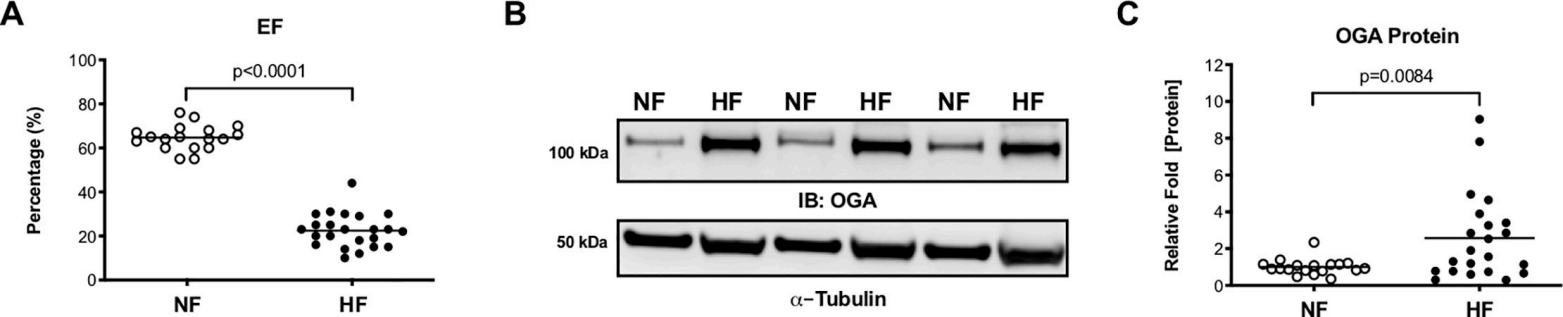
OGA is upregulated in human heart failure. Cardiac tissue from non-failing (NF, n = 18) and failing hearts (HF, n = 23) was used to assess the expression of OGA. Left ventricular ejection fraction (A). Western blot of OGA protein expression (B). Densitometric analysis of OGA western (C). An unpaired Student’s *t*-test was used to determine significance between NF and HF groups.

We tested whether O-GlcNAc metabolizing enzymes were changed in failing human hearts (see S6 Table for demographics). Patients with heart failure had significantly lower ejection fraction than patients without heart failure (Fig 5A). Failing hearts had higher protein expression of OGA compared to non-failing tissue (Fig 5B and 5C). Overall protein O-GlcNAcylation was not changed between non failing and failing hearts (S7 Fig). These data combined with our previously published data [33] recapitulate the notion that dysregulation of O-GlcNAc metabolizing enzymes occurs in human heart failure.

## Discussion

Our previous work indicated the requirement for *Ogt* following infarct-induced heart failure [19]. Our observation that OGT promoted an increase in pro-adaptive O-GlcNAcylation was consonant with our previous insights, albeit in acute model systems. In the present study, we tested the complementary hypothesis that reduction in cardiomyocyte OGA expression would increase O-GlcNAc levels and attenuated the severity of heart failure. We also established an inducible, cardiomyocyte-specific, OGA-deficient mouse. Under naïve conditions, *Oga* deficiency slightly reduced cardiac ejection fraction. Contrary to our prediction, diminution of OGA expression exacerbated cardiac dysfunction after MI. *Oga* haploinsufficient hearts had worse cardiac dysfunction within 1 wk of MI without changes to cardiac remodeling. This initial exacerbation in cardiac dysfunction was not evident at 4 wk post-MI. Interestingly, differences in cardiomyocyte size were evident in *Oga*-deficient hearts; cardiomyocytes in the remote zone were larger and those in the infarct zone were smaller compared to the *Oga*-competent hearts. Thus, perturbation of either *Ogt* or *Oga* (i.e., OGA) hastens the severity of infarct-induced heart failure.

It is worth noting that despite similarities between our mouse and human data, there were qualitative differences between the two species. Here and previously [19], we found that downregulation of OGA expression occurs following infarct-induced heart failure in mice. Conversely, OGA expression in human heart failure samples is also elevated, not suppressed, as we see in our chronic mouse model [31]. Despite this difference, both human and mouse heart failure tissue are characterized by alterations in O-GlcNAcylation [19, 31, 33, 36, 37]. The levels of OGT and OGA are highly regulated by diverse mechanisms including polycomb repression [38], microRNAs [31], OGT intron retention [39], and HBP (hexosamine biosynthetic pathway) flux [40]. The peripheral question of why there is a difference in OGA expression in failing mouse versus human hearts remains. There is a component of OGA cycling during the adaptive response that could reconcile differences of expression. In addition, it is possible that the relative age differences (the humans were much older) may figure in this observation. It is also possible that the duration (longer in humans) or severity (more in mice) of heart failure may also influence this differential response. It is important to highlight that the human samples came from humans who were diabetic—this is a major difference. Lastly, heart failure patients are medically managed whereas our preclinical models do not receive a human standard of care (no beta-blockers, ACE inhibitors, etc).

So, how do we reconcile our present results with our previous complementary study in which we ablated OGT in cardiomyocytes? That is, in our previous investigation of OGT deletion in heart failure, we found that the mice did worse—similar to the present observations. Without careful consideration of the aggregate results of the present work focusing on OGA haploinsufficiency and our previous work focusing on OGT deletion [19], it would seem that the two studies conflict with one another. Indeed, if O-GlcNAcylation were a static event this might be a reasonable conclusion; however, O-GlcNAcylation is dynamic. There are also compensatory changes in the levels of O-GlcNAc cycling enzymes which are tissue specific and dependent upon metabolic status [1]. The presence/absence of O-GlcNAc likely changes—in fact, it could be argued that the real issue is occupancy (i.e., the relative presence/absence of O-GlcNAc at a given site). Such a consideration requires acknowledgement of the possibility that O-GlcNAc cycles at sites—not simply present or absent at a site for the life of a protein. That OGT deletion and OGA deletion phenocopy one another is not without precedence. In *C*. *elegans*, deletion of *ogt-1(ok430)* or *oga-1(ok1207)* resulted in similar alterations in metabolism, macronutrient storage and intracellular signaling [41, 42]. Thus, we speculate that O-GlcNAc cycling, rather than simple presence or absence, is critical for pro-adaptive changes in the failing heart. Thus, loss of OGT or loss of OGA create the same result in chronic conditions (at least in heart failure) because both interventions block cycling of O-GlcNAc, despite having opposing effects on steady state O-GlcNAc levels. Although the present study does not directly address this speculation, the data support this contention as a new hypothesis to be tested in future studies.

There are several unanswered questions regarding how OGT, OGA, and O-GlcNAcylation are regulated. Little work has been done in this area; however, some insights have emerged. We have identified candidates that may regulate OGT and OGA expression: miRNA-539 [31] and the transcription factor E2F1 [43]. MiRNA-539 is elevated in HF and its upregulation coincides with suppression of OGA. Moreover, miRNA-539 was predicted to target *Oga* mRNA. Through a series of reporter assays we provided evidence that miRNA-539 negative regulates OGA expression *in vitro* [31]. In a separate study, we demonstrated that E2F1 negatively regulates OGT and OGA expression by binding to presumptive promoter regions of *Ogt* and *Oga* [43]. Despite these being the first studies to identify molecular regulators of OGT and OGA expression, it is not clear whether these observations extended to the intact heart. Recently, we tested whether deletion of *E2f1* could attenuate post-infarction ventricular remodeling by alleviating repression of O-GlcNAc cycling enzymes [44]. Although deletion of *E2f1* did attenuate ventricular remodeling, it did not exert an appreciable effect on O-GlcNAcylation. Thus, the regulation of O-GlcNAcylation in the failing heart remains enigmatic and is likely to be multifactorial.

Although the O-GlcNAc modification does not occur via a canonical consensus sequence, many proteins harboring this modification have been identified; however, the level of evidence for such O-GlcNAc modified proteins (particularly site-specific identities) varies widely. To determine whether a specific O-GlcNAc site is responsible for a given effect on the function of a protein, a classical approach would be to perform site-directed mutagenesis. Unfortunately, because the O-GlcNAc modification occurs on serine/threonine residues, investigators are often frustrated by the potential impact (even if only theoretical) of interfering with phosphorylation—a concern that does not seem to affect investigators of phosphorylation sites (i.e., ignoring potential inadvertent interference with O-GlcNAcylation). One may still wonder why the present study did not focus on the protein targets of O-GlcNAcylation that could explain the observed effects. Although this may be fruitful in limited circumstances, the more immediate need was to understand the overarching phenomenon before becoming lost in the minutiae. Thus, our approach was to take a system-wide view to determine whether simple increases or decreases in O-GlcNAcylation predicted (or were associated with) the outcome in heart failure. Indeed, had the reduced-function OGA mouse (with increased O-GlcNAc levels) shown improvement in EF during heart failure, the urgency to identify protein targets would be clear; however, we observed that either deletion of *Ogt* [19] or reduction of *Oga*—which have opposite effects on global O-GlcNAc levels—culminates in exacerbation of heart failure. In our model, haploinsufficiency of *Oga* was sufficient to affect cardiac function even in naïve conditions following extended observation. Interestingly, there is a link between excessive O-GlcNAcylation and cardiac dysfunction [45]. Indeed, overexpressing OGA was shown to ameliorate cardiac dysfunction in diabetic mice with elevated O-GlcNAc levels [45]. In contrast, our HF study demonstrated *Oga*-deficient mice with significantly elevated O-GlcNAc levels displayed no further exacerbation in cardiac function after 1 wk. These collective observations suggest that identifying specific O-GlcNAc-modified protein targets may not be the most proximal question to address.

The results of these studies question the uncomplicated view of simply directionally changing O-GlcNAc levels, i.e., more or less O-GlcNAc is good or bad. Instead, our previous and present work provide evidence that blocking the ability of cycling O-GlcNAc exacerbates heart failure. It is possible that O-GlcNAcylation, like a thermostat, requires the ability to constantly change. In other words, O-GlcNAcylation can be temporarily increased or decreased; however, blocking either side of the equation for extended periods of time likely disrupts homeostasis, or, given sufficient time, may actually serve as the nidus for disease.

## Supporting information

S1 Fig Generation of icm*Oga* ablated mice. Genotyping results of wild-type and heterogenous *Oga* floxed mice (A). PCR products of the floxed *Oga* appear at 850 bp and the wild-type *Oga* PCR product appears at 792 bp. To generate mice capable of tamoxifen-induced, cardiomyocyte-specific, heterozygous deletion of *Oga* (icm*Oga*^*+/*^), we crossed *Oga*^*fl/+*^ *with* MCM^+/-^ mice to generate *Oga*^*fl/+*^ MCM^+/-^ mice (B). The *Oga* floxed strain was described previously [30]. Timeline for tamoxifen treatment (C). Mice were treated with with 20 mg/kg of tamoxifen IP for 5 d consecutively. Tamoxifen was allowed to washout for 5 d. Baseline echo was performed at day 11. Mice were subjected to MI 1 wk later.
(TIFF)

S2 Fig Induced OGA haploinsufficiency is limited to the heart. To determine if ablation of OGA was limited to the heart we isolated protein from skeletal muscle, lung, and kidney from icm*Oga*^+/+^ and icm*Oga*^*+/-*^ mice. Immunoblot for OGA protein in skeletal muscle (A), lung (B), and kidney (C) tissue.
(TIFF)

S3 Fig Cardiomyocyte size and capillary density are unaffected by reduction of cardiac *Oga* 1 wk post-MI. Gene expression of markers of cardiac hypertrophy 1 wk post-MI(A). Gravimetric analysis of heart size; heart weight to tibia length (B) Representative images of WGA and isolectin-stained icm*Oga*^*+/-*^ and icm*Oga*^*+/+*^ heart sections (C). Cardiomyocyte cross-sectional area was measured in the area bordering the infarct (BZ), in the infarct zone (IZ), and remote (RZ) from the infarct (D). Capillary density in border (BZ), infarct (IZ), and remote zone (RZ) (E). An unpaired Student’s *t*-test was used to determine significance between icm*Oga*^*+/-*^ and icm*Oga*^*+/+*^groups.
(TIFF)

S4 Fig *Oga* deficiency does not affect apoptosis 1 wk post MI. Gene expression of apoptosis marker *Bcl2* (A). Representative TUNEL-stained sections (B). Quantification of TUNEL positive cells (C). An unpaired Student’s *t*-test was used to determine significance between icm*Oga*^*+/-*^ and icm*Oga*^*+/+*^groups.
(TIFF)

S5 Fig OGA deficiency hastens HF early after MI. A separate cohort of mice was subjected to extended observation after 1 wk MI. Cardiac function of the left ventricle was assessed at both 1 and 4 wk post-MI. Left ventricle Ejection fraction was significantly lower at 1 wk in the icmOga^+/-^ mice. By 4 wk, the ejection fraction of the icm*Oga*^+/+^ mice resembled that of the OGA-deficient mice (A). An unpaired Student’s *t*-test was used to determine significance between icm*Oga*^+/+^ and icm*Oga*^*+/-*^ groups.
(TIFF)

S6 Fig *Oga* deficiency does not affect apoptosis 4 wk-post MI. Gene expression of apoptosis marker *Bcl2* (A). Representative TUNEL-stained sections (B). Quantification of TUNEL positive cells (C). An unpaired Student’s *t*-test was used to determine significance between icm*Oga*^*+/-*^ and icm*Oga*^*+/+*^groups.
(TIFF)

S7 Fig O-GlcNAcylation is preserved in human heart failure. Cardiac tissue from non-failing (NF, n = 18) and failing hearts (HF, n = 23) was used to assess the expression of overall O-GlcNAcylation. Western blot of protein O-GlcNAcylation (A) and subsequent densitometric analysis (B). An unpaired Student’s *t*-test was used to determine significance between NF and HF groups.
(TIFF)

S1 Table Genotyping primers. Primer sequences used for genotyping *Oga* floxed and MCM mice.
(TIFF)

S2 Table RT-PCR primers. Primer sequences used for RT-PCR.
(TIFF)

S3 Table Reduction in *Oga* does not promote cardiac dysfunction in naïve mice. Naïve, 10–18 wk-old male and female icm*Oga*^+/-^ (n = 10; 6 were male and 4 were female) and their icm*Oga*^+/+^ (n = 10; 7 were male and 3 were female) littermates treated with tamoxifen were subjected to echocardiography. Cardiac function of the left ventricle was assessed. Naïve icm*Oga*^*+/-*^ mice demonstrated a lower left ventricular ejection fraction (EF) compared to icm*Oga*^+/+^ mice. No changes in left ventricular end-diastolic volume (EDV), left ventricular end-systolic volume (ESV), heart rate (HR), stroke volume (SV), cardiac output (CO), left ventricular inner systolic diameter (LVIDs), left ventricular inner diastolic diameter (LVIDd), fractional shortening (FS), left ventricular posterior wall thickness in diastole (LVPWd), left ventricular posterior wall thickness in systole (LVPWs), and left ventricular anterior wall thickness in diastole or systole (LVAWd, LVAWs). An unpaired Student’s *t*-test was used to determine significance between icm*Oga*^+/+^ and icm*Oga*^*+/-*^ groups.
(TIFF)

S4 Table Reduction of cardiac OGA does not affect cardiac function in female mice 1 wk-post MI. Tamoxifen-treated female icm*Oga*^+/+^ (n = 13) and icm*Oga*^+/-^ (n = 13) were subjected to echocardiography after 1 wk post-MI. Cardiac function of the left ventricle was assessed. No changes were observed in left ventricular end-diastolic volume (EDV), left ventricular end-systolic volume (ESV), ejection fraction (EF), heart rate (HR), stroke volume (SV), cardiac output (CO), left ventricular inner systolic diameter (LVIDs), left ventricular inner diastolic diameter (LVIDd), fractional shortening (FS), left ventricular posterior wall thickness in diastole (LVPWd), left ventricular posterior wall thickness in systole (LVPWs), and diastolic or systolic left ventricular anterior wall thickness in diastole or systole (LVAWd, LVAWs). An unpaired Student’s *t*-test was used to determine significance between icm*Oga*^+/+^ and icm*Oga*^*+/-*^ groups.
(TIFF)

S5 Table Cardiac function was unchanged at 4 wk post-MI in OGA deficient mice. Tamoxifen-treated male icm*Oga*^+/+^ (n = 9) and icm*Oga*^+/-^ (n = 7) were subjected to echocardiography after 4 wk post-MI. Cardiac function of the left ventricle was assessed. No changes were observed in left ventricular end-diastolic volume (EDV), left ventricular end-systolic volume (ESV), left ventricular ejection fraction (EF), heart rate (HR), stroke volume (SV), cardiac output (CO), left ventricular inner systolic diameter (LVIDs), left ventricular inner diastolic diameter (LVIDd), fractional shortening (FS), left ventricular posterior wall thickness in diastole (LVPWd), left ventricular posterior wall thickness in systole (LVPWs), and diastolic or systolic left ventricular anterior wall thickness in diastole or systole (LVAWd, LVAWs). An unpaired Student’s *t*-test was used to determine significance between icm*Oga*^+/+^ and icm*Oga*^*+/-*^ groups.
(TIFF)

S6 Table Patient demographics. Patient demographics of de-identified human samples. A chi-squared test was used to determined significance between NF and HF demographics.
(TIFF)

S1 Raw images (PDF)

## Abbreviations

HBP: hexosamine biosynthetic pathway
MI: myocardial infarction
O-GlcNAc: β-O-linked N-acetylglucosamine
OGA: O-GlcNAcase
OGT: O-GlcNAc transferase
