# Supplementary figures and images for: Cardiomyocyte Oga haploinsufficiency increases O-GlcNAcylation but hastens ventricular dysfunction following myocardial infarction
Source: PLoS One. 2020 Nov 30;15(11):e0242250. doi: 10.1371/journal.pone.0242250 (PMC7703924; doi:10.1371/journal.pone.0242250)

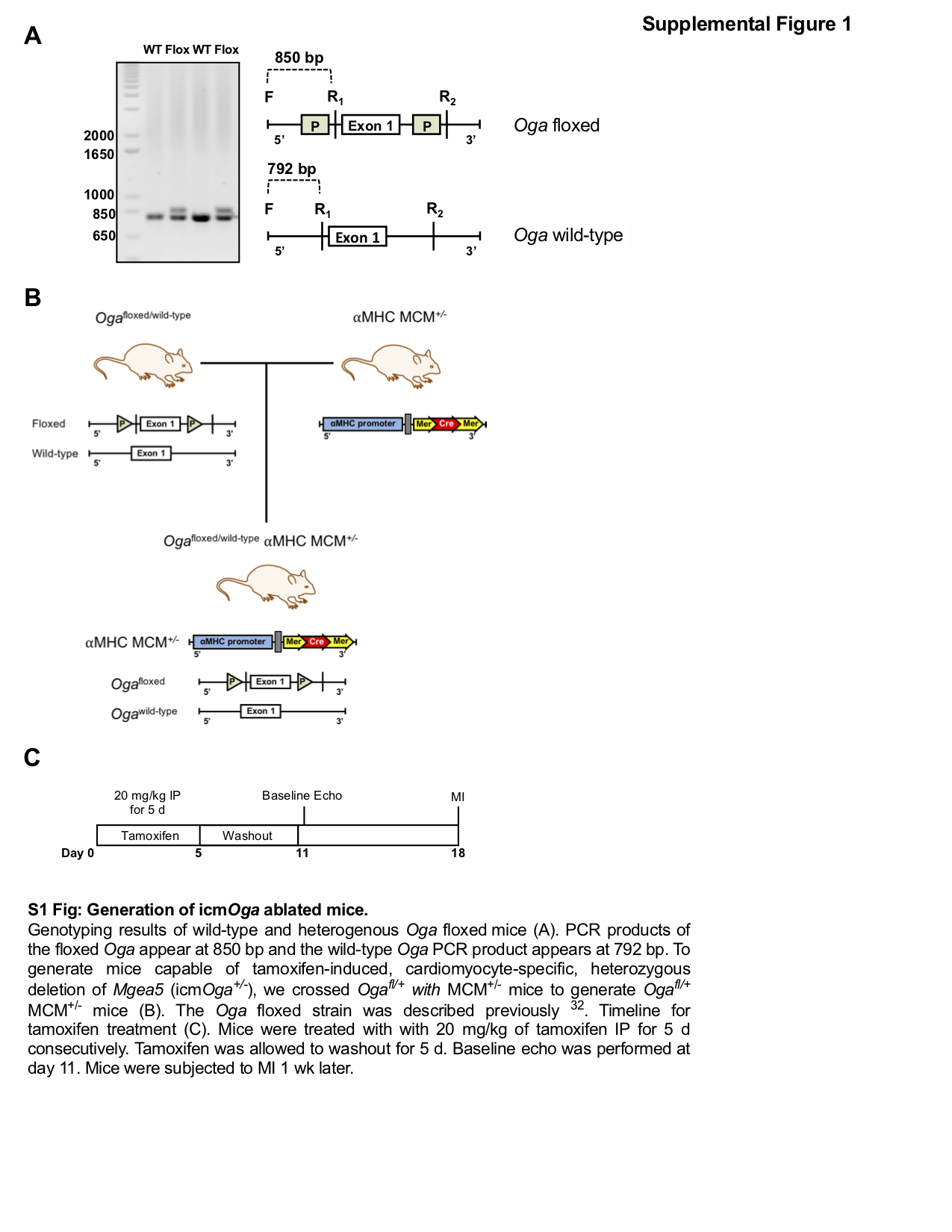

Supplement: S1 Fig — Genotyping results of wild-type and heterogenous Oga floxed mice (A). PCR products of the floxed Oga appear at 850 bp and the wild-type Oga PCR product appears at 792 bp. To generate mice capable of tamoxifen-induced, cardiomyocyte-specific, heterozygous deletion of Oga (icmOga+/), we crossed Ogafl/+ with MCM+/- mice to generate Ogafl/+ MCM+/- mice (B). The Oga floxed strain was described previously [30]. Timeline for tamoxifen treatment (C). Mice were treated with with 20 mg/kg of tamoxifen IP for 5 d consecutively. Tamoxifen was allowed to washout for 5 d. Baseline echo was performed at day 11. Mice were subjected to MI 1 wk later. (TIFF) [file pone.0242250.s001.tiff]

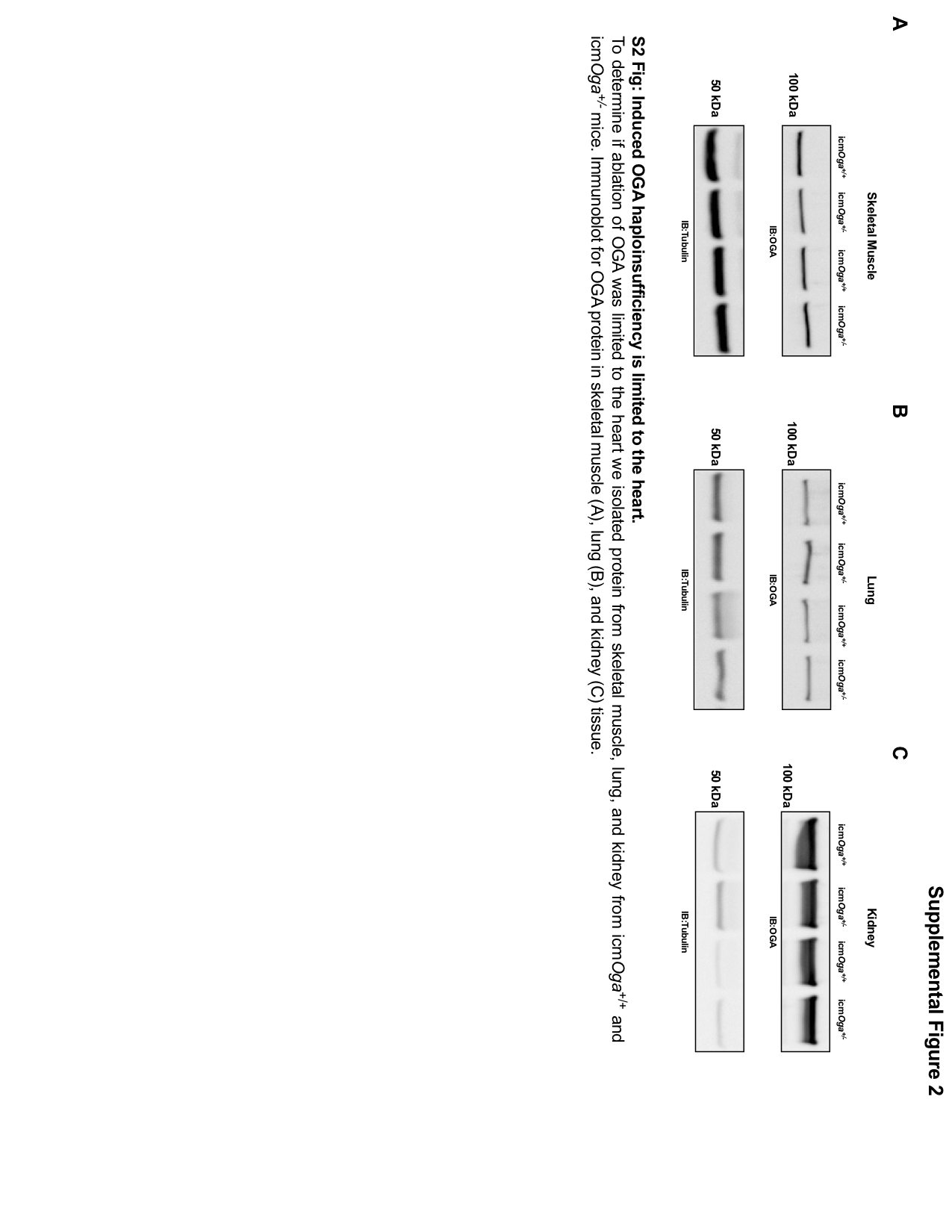

Supplement: S2 Fig — To determine if ablation of OGA was limited to the heart we isolated protein from skeletal muscle, lung, and kidney from icmOga+/+ and icmOga+/- mice. Immunoblot for OGA protein in skeletal muscle (A), lung (B), and kidney (C) tissue. (TIFF) [file pone.0242250.s002.tiff]

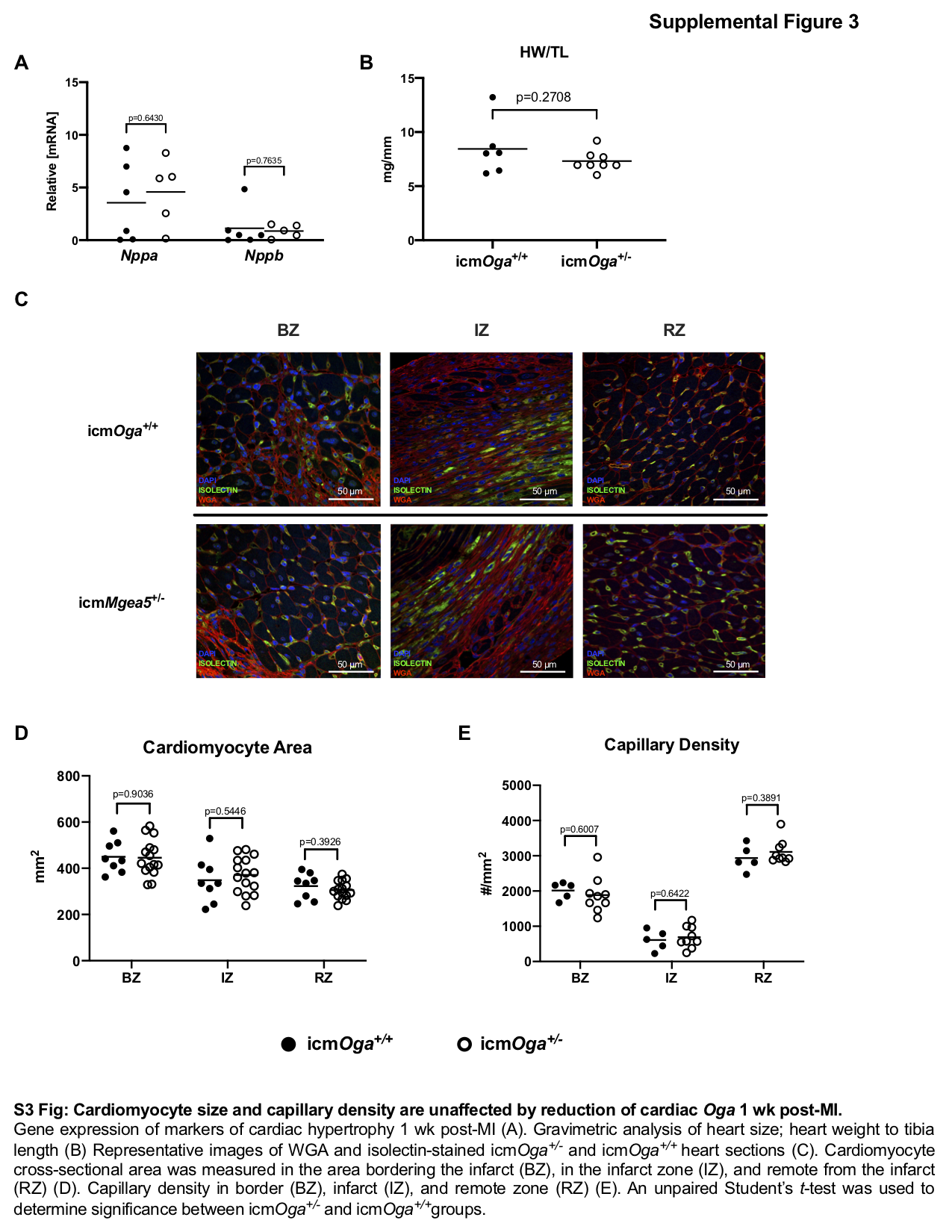

Supplement: S3 Fig — Gene expression of markers of cardiac hypertrophy 1 wk post-MI(A). Gravimetric analysis of heart size; heart weight to tibia length (B) Representative images of WGA and isolectin-stained icmOga+/- and icmOga+/+ heart sections (C). Cardiomyocyte cross-sectional area was measured in the area bordering the infarct (BZ), in the infarct zone (IZ), and remote (RZ) from the infarct (D). Capillary density in border (BZ), infarct (IZ), and remote zone (RZ) (E). An unpaired Student’s t-test was used to determine significance between icmOga+/- and icmOga+/+groups. (TIFF) [file pone.0242250.s003.tiff]

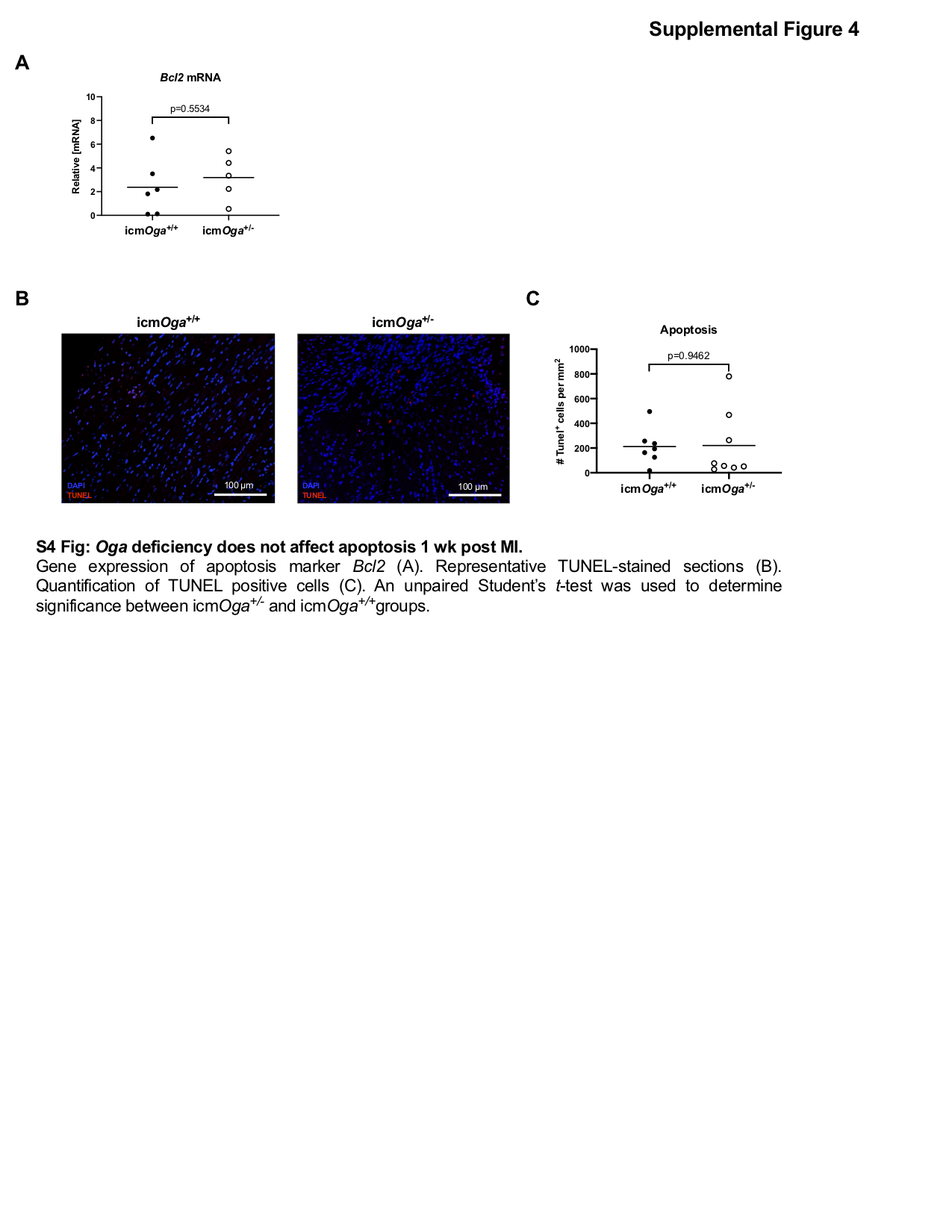

Supplement: S4 Fig — Gene expression of apoptosis marker Bcl2 (A). Representative TUNEL-stained sections (B). Quantification of TUNEL positive cells (C). An unpaired Student’s t-test was used to determine significance between icmOga+/- and icmOga+/+groups. (TIFF) [file pone.0242250.s004.tiff]

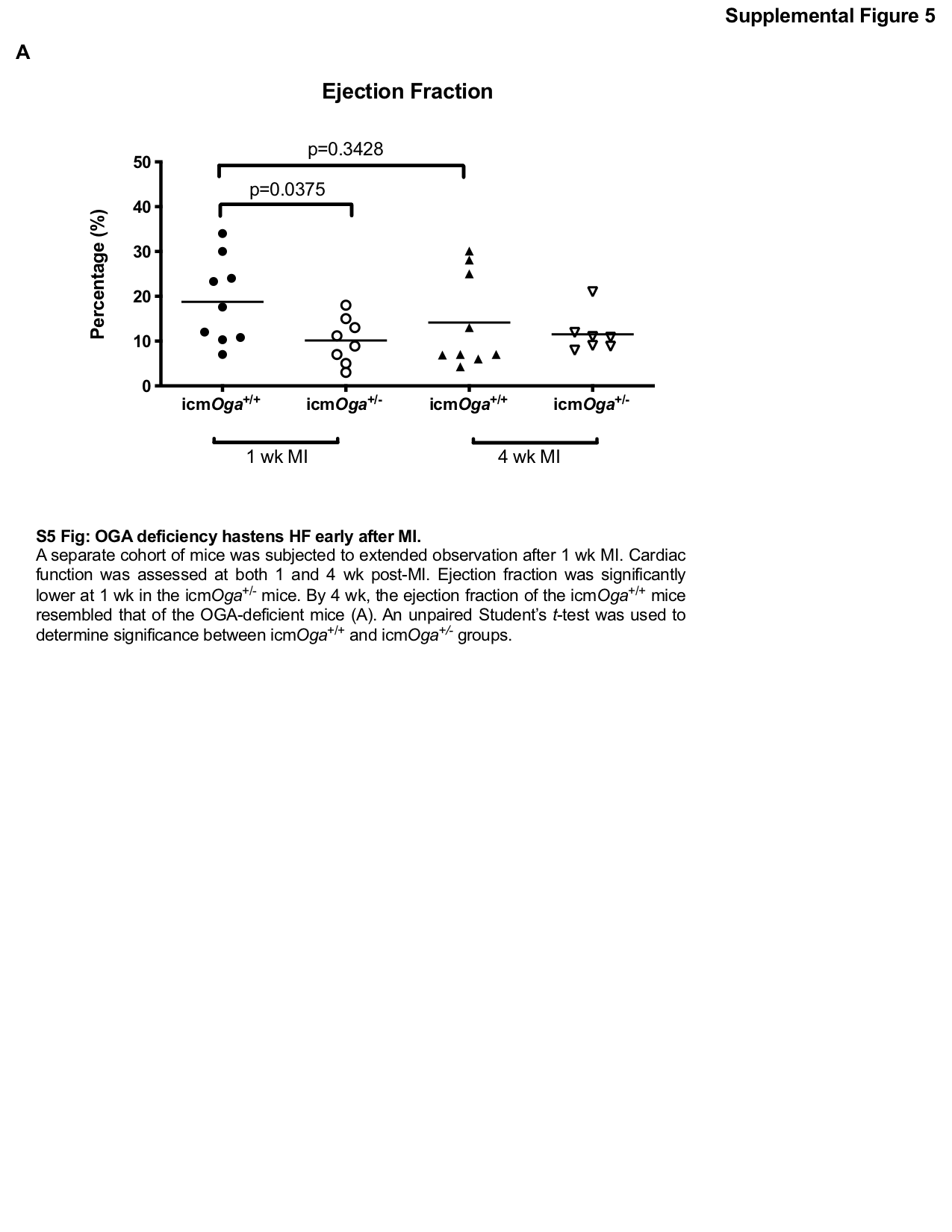

Supplement: S5 Fig — A separate cohort of mice was subjected to extended observation after 1 wk MI. Cardiac function of the left ventricle was assessed at both 1 and 4 wk post-MI. Left ventricle Ejection fraction was significantly lower at 1 wk in the icmOga+/- mice. By 4 wk, the ejection fraction of the icmOga+/+ mice resembled that of the OGA-deficient mice (A). An unpaired Student’s t-test was used to determine significance between icmOga+/+ and icmOga+/- groups. (TIFF) [file pone.0242250.s005.tiff]

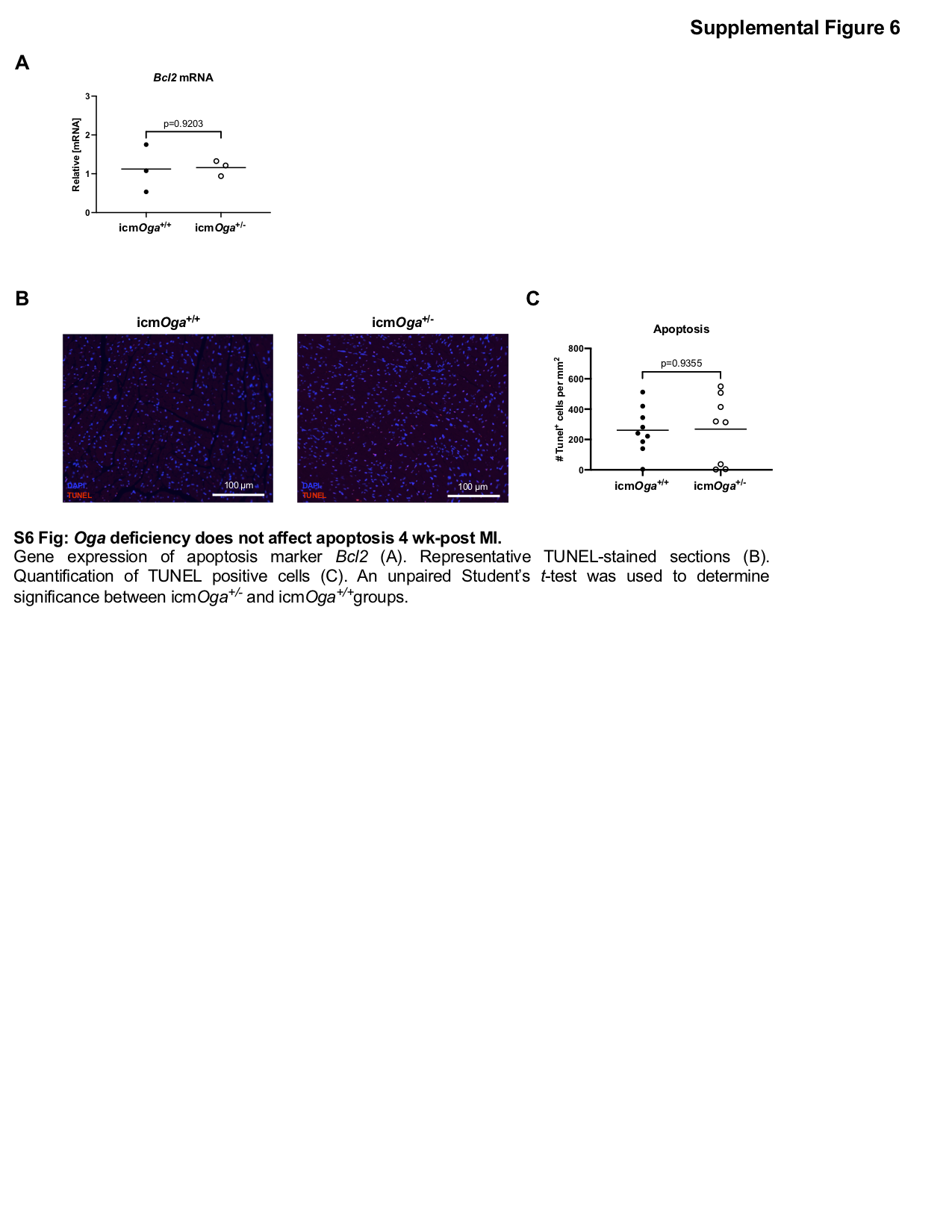

Supplement: S6 Fig — Gene expression of apoptosis marker Bcl2 (A). Representative TUNEL-stained sections (B). Quantification of TUNEL positive cells (C). An unpaired Student’s t-test was used to determine significance between icmOga+/- and icmOga+/+groups. (TIFF) [file pone.0242250.s006.tiff]

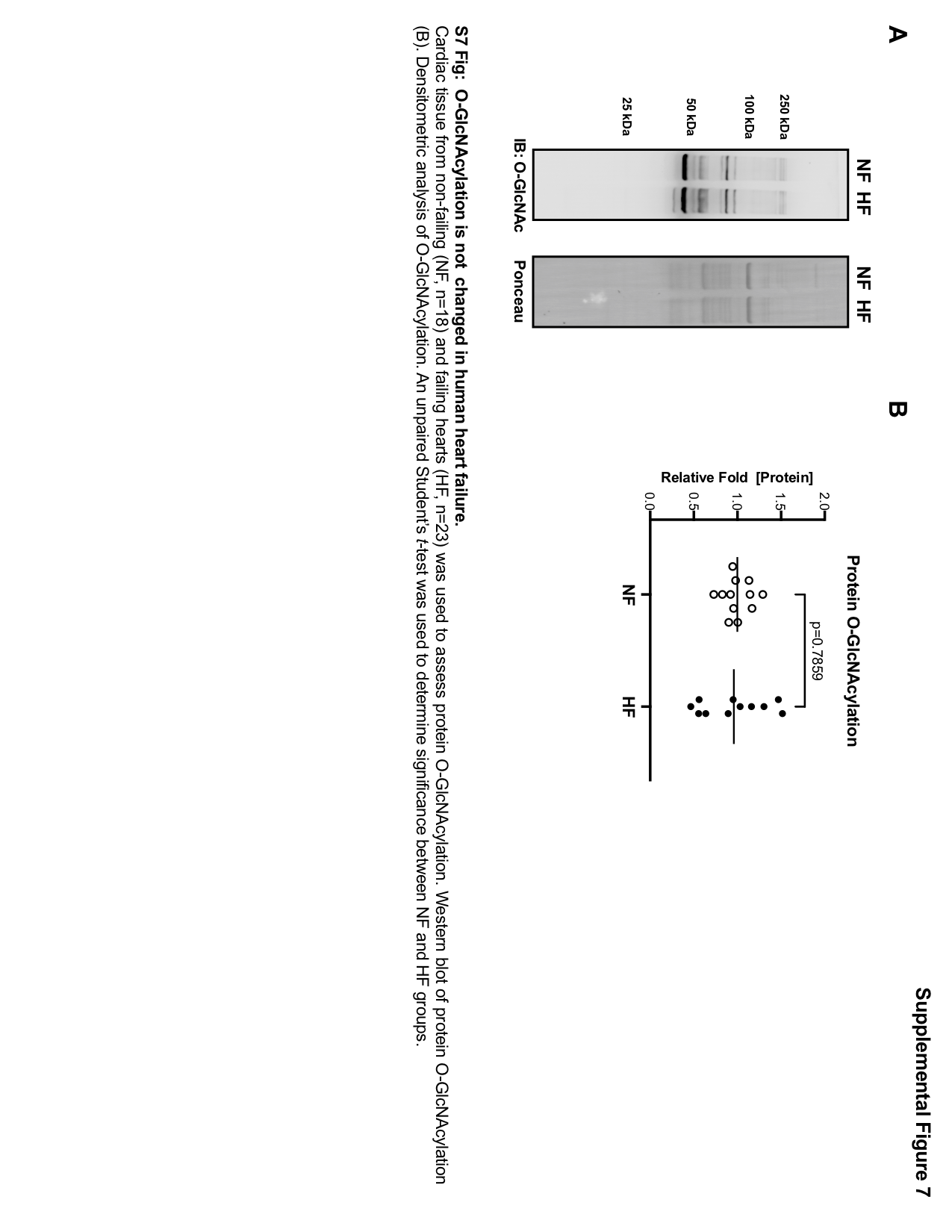

Supplement: S7 Fig — Cardiac tissue from non-failing (NF, n = 18) and failing hearts (HF, n = 23) was used to assess the expression of overall O-GlcNAcylation. Western blot of protein O-GlcNAcylation (A) and subsequent densitometric analysis (B). An unpaired Student’s t-test was used to determine significance between NF and HF groups. (TIFF) [file pone.0242250.s007.tiff]

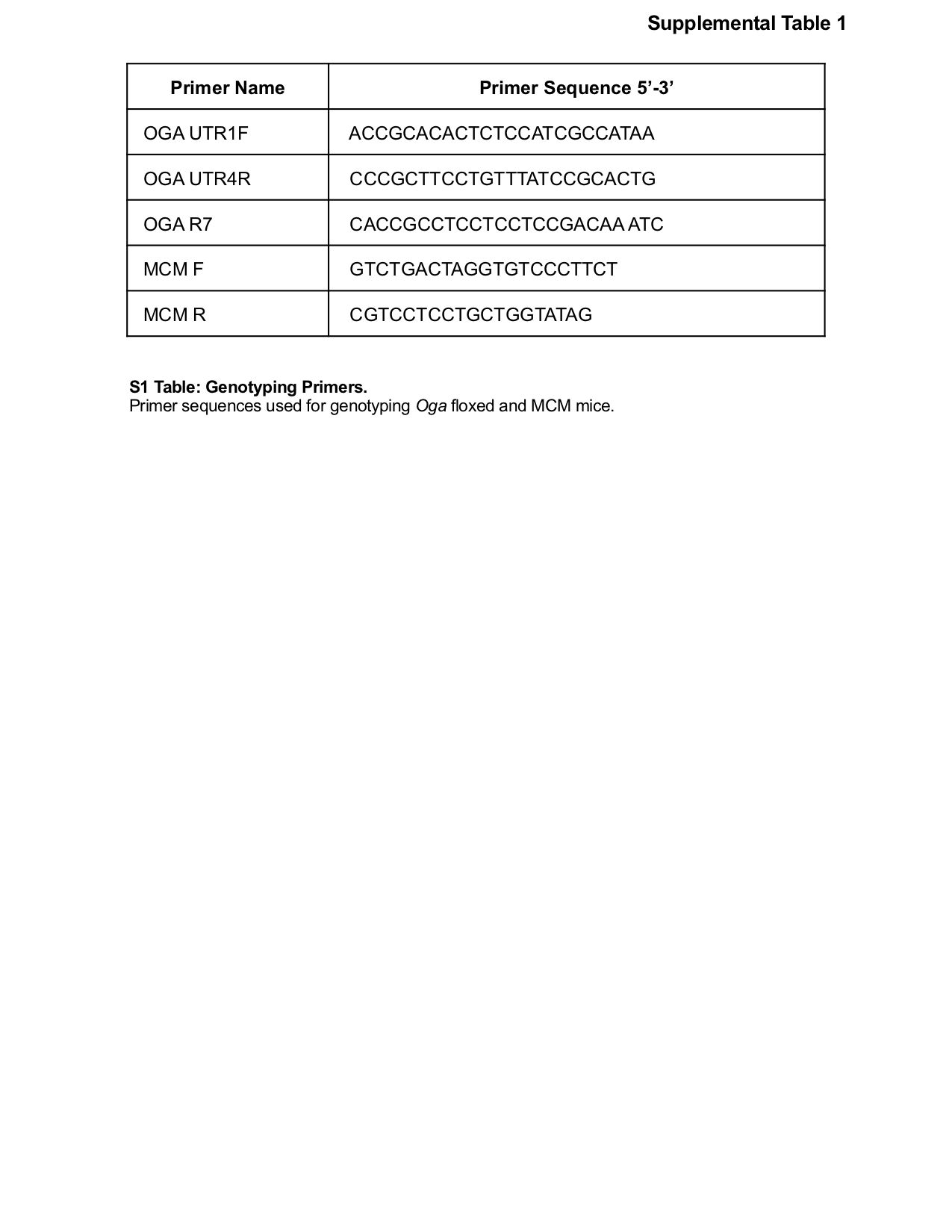

Supplement: S1 Table — Primer sequences used for genotyping Oga floxed and MCM mice. (TIFF) [file pone.0242250.s008.tiff]

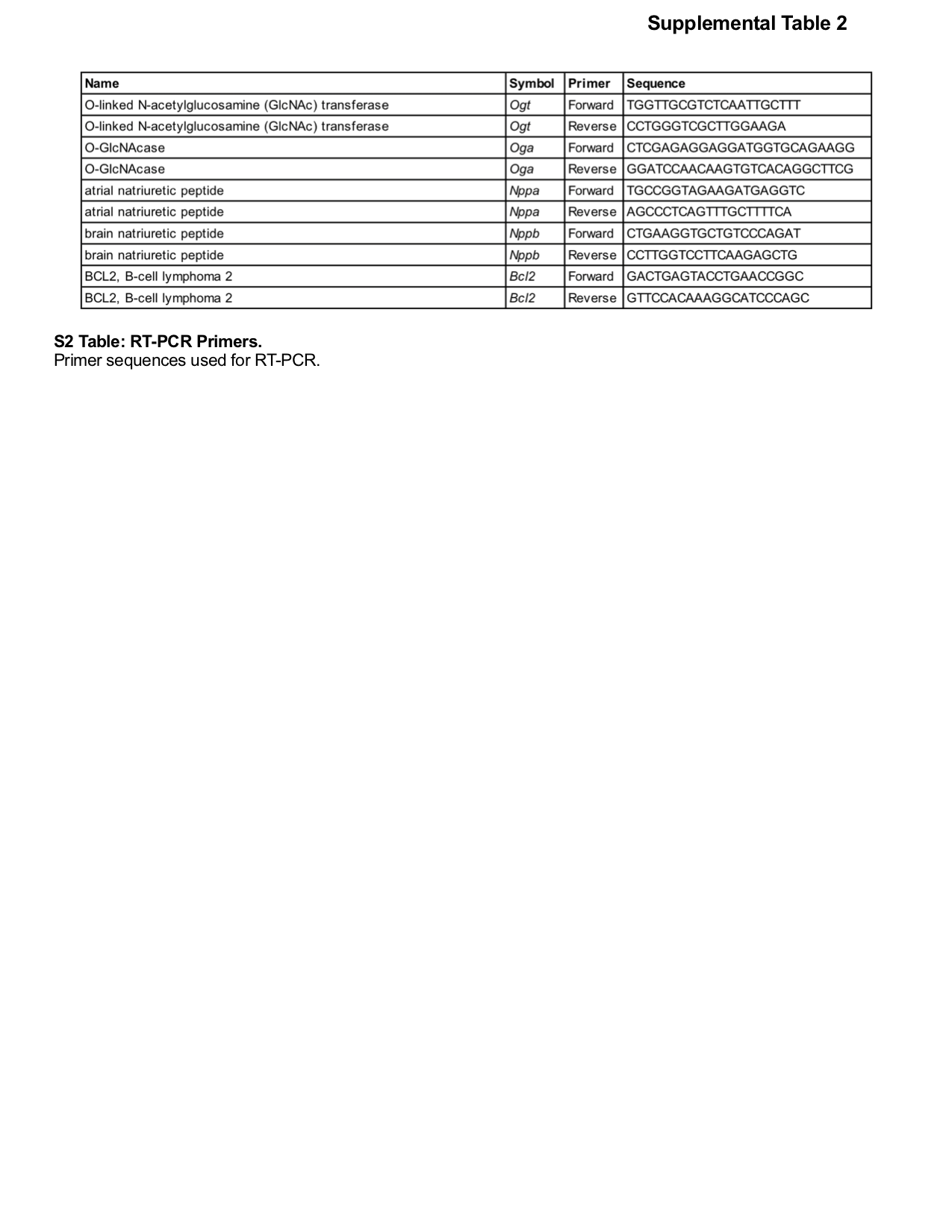

Supplement: S2 Table — Primer sequences used for RT-PCR. (TIFF) [file pone.0242250.s009.tiff]

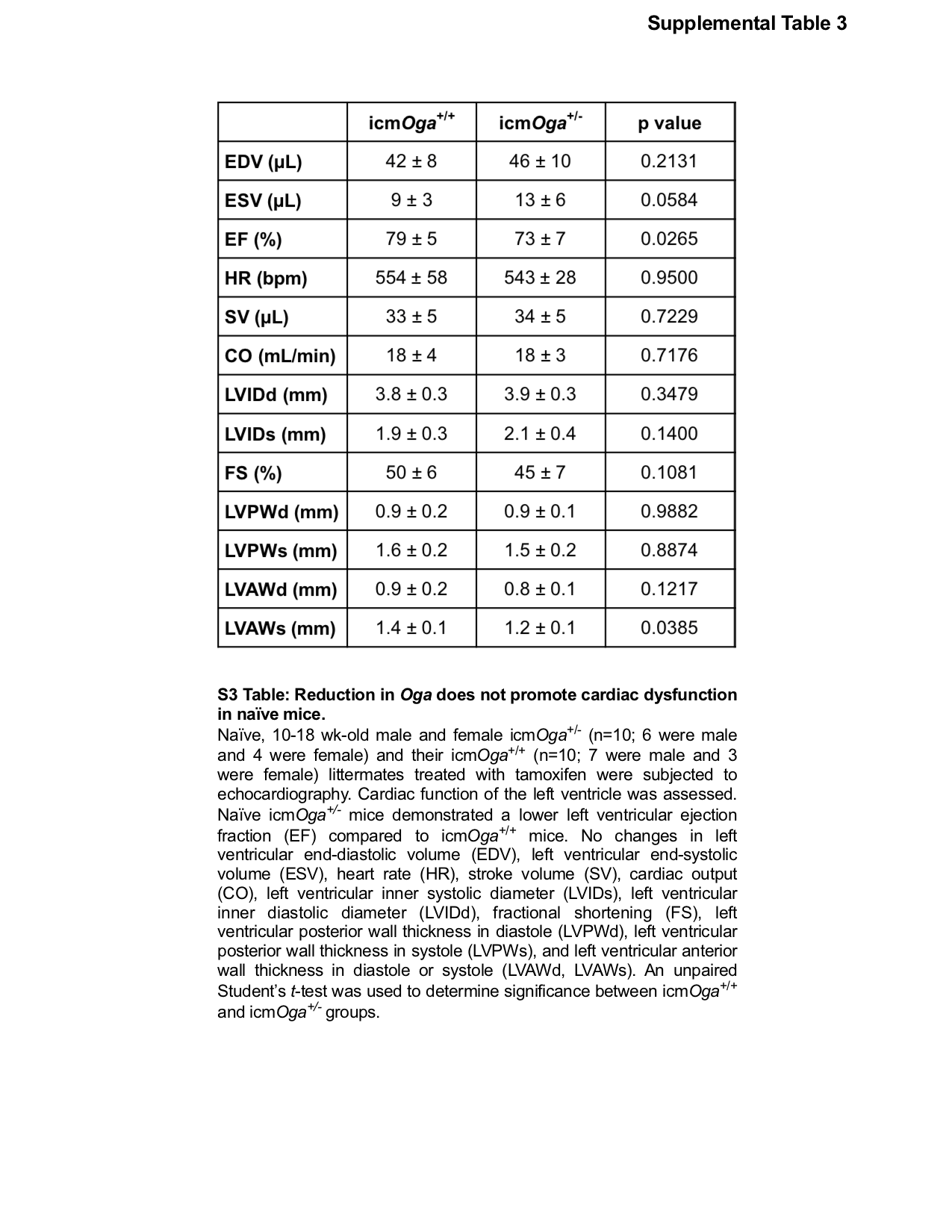

Supplement: S3 Table — Naïve, 10–18 wk-old male and female icmOga+/- (n = 10; 6 were male and 4 were female) and their icmOga+/+ (n = 10; 7 were male and 3 were female) littermates treated with tamoxifen were subjected to echocardiography. Cardiac function of the left ventricle was assessed. Naïve icmOga+/- mice demonstrated a lower left ventricular ejection fraction (EF) compared to icmOga+/+ mice. No changes in left ventricular end-diastolic volume (EDV), left ventricular end-systolic volume (ESV), heart rate (HR), stroke volume (SV), cardiac output (CO), left ventricular inner systolic diameter (LVIDs), left ventricular inner diastolic diameter (LVIDd), fractional shortening (FS), left ventricular posterior wall thickness in diastole (LVPWd), left ventricular posterior wall thickness in systole (LVPWs), and left ventricular anterior wall thickness in diastole or systole (LVAWd, LVAWs). An unpaired Student’s t-test was used to determine significance between icmOga+/+ and icmOga+/- groups. (TIFF) [file pone.0242250.s010.tiff]

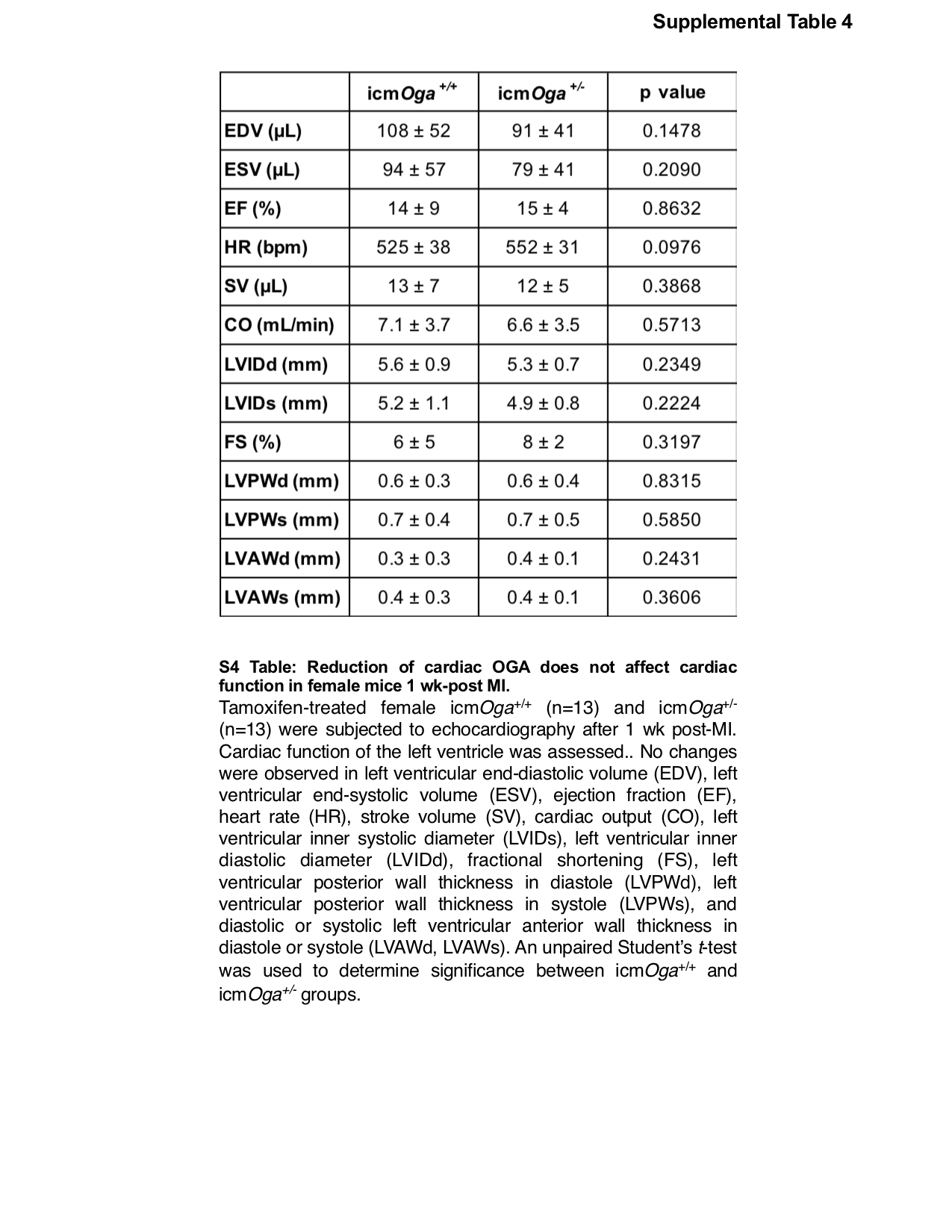

Supplement: S4 Table — Tamoxifen-treated female icmOga+/+ (n = 13) and icmOga+/- (n = 13) were subjected to echocardiography after 1 wk post-MI. Cardiac function of the left ventricle was assessed. No changes were observed in left ventricular end-diastolic volume (EDV), left ventricular end-systolic volume (ESV), ejection fraction (EF), heart rate (HR), stroke volume (SV), cardiac output (CO), left ventricular inner systolic diameter (LVIDs), left ventricular inner diastolic diameter (LVIDd), fractional shortening (FS), left ventricular posterior wall thickness in diastole (LVPWd), left ventricular posterior wall thickness in systole (LVPWs), and diastolic or systolic left ventricular anterior wall thickness in diastole or systole (LVAWd, LVAWs). An unpaired Student’s t-test was used to determine significance between icmOga+/+ and icmOga+/- groups. (TIFF) [file pone.0242250.s011.tiff]

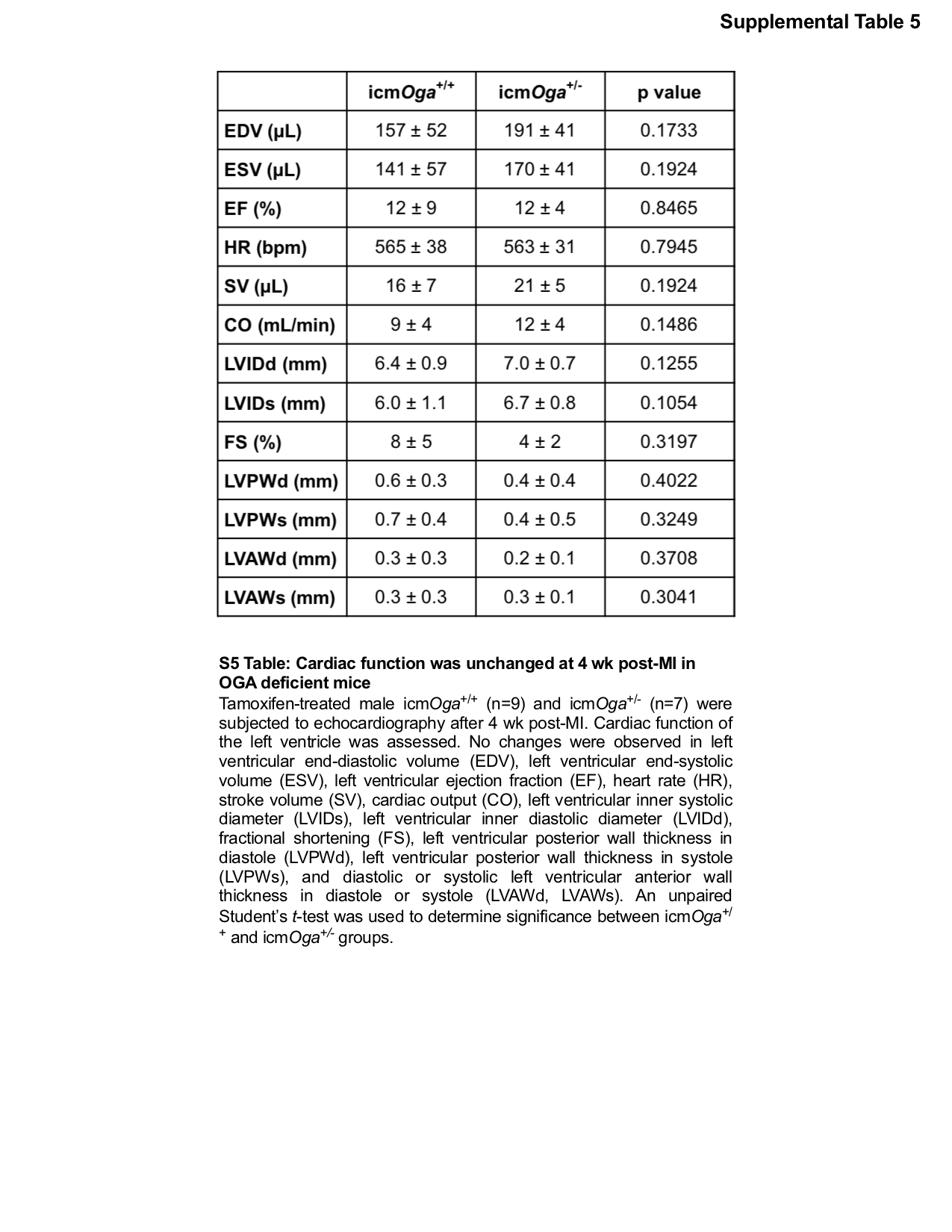

Supplement: S5 Table — Tamoxifen-treated male icmOga+/+ (n = 9) and icmOga+/- (n = 7) were subjected to echocardiography after 4 wk post-MI. Cardiac function of the left ventricle was assessed. No changes were observed in left ventricular end-diastolic volume (EDV), left ventricular end-systolic volume (ESV), left ventricular ejection fraction (EF), heart rate (HR), stroke volume (SV), cardiac output (CO), left ventricular inner systolic diameter (LVIDs), left ventricular inner diastolic diameter (LVIDd), fractional shortening (FS), left ventricular posterior wall thickness in diastole (LVPWd), left ventricular posterior wall thickness in systole (LVPWs), and diastolic or systolic left ventricular anterior wall thickness in diastole or systole (LVAWd, LVAWs). An unpaired Student’s t-test was used to determine significance between icmOga+/+ and icmOga+/- groups. (TIFF) [file pone.0242250.s012.tiff]

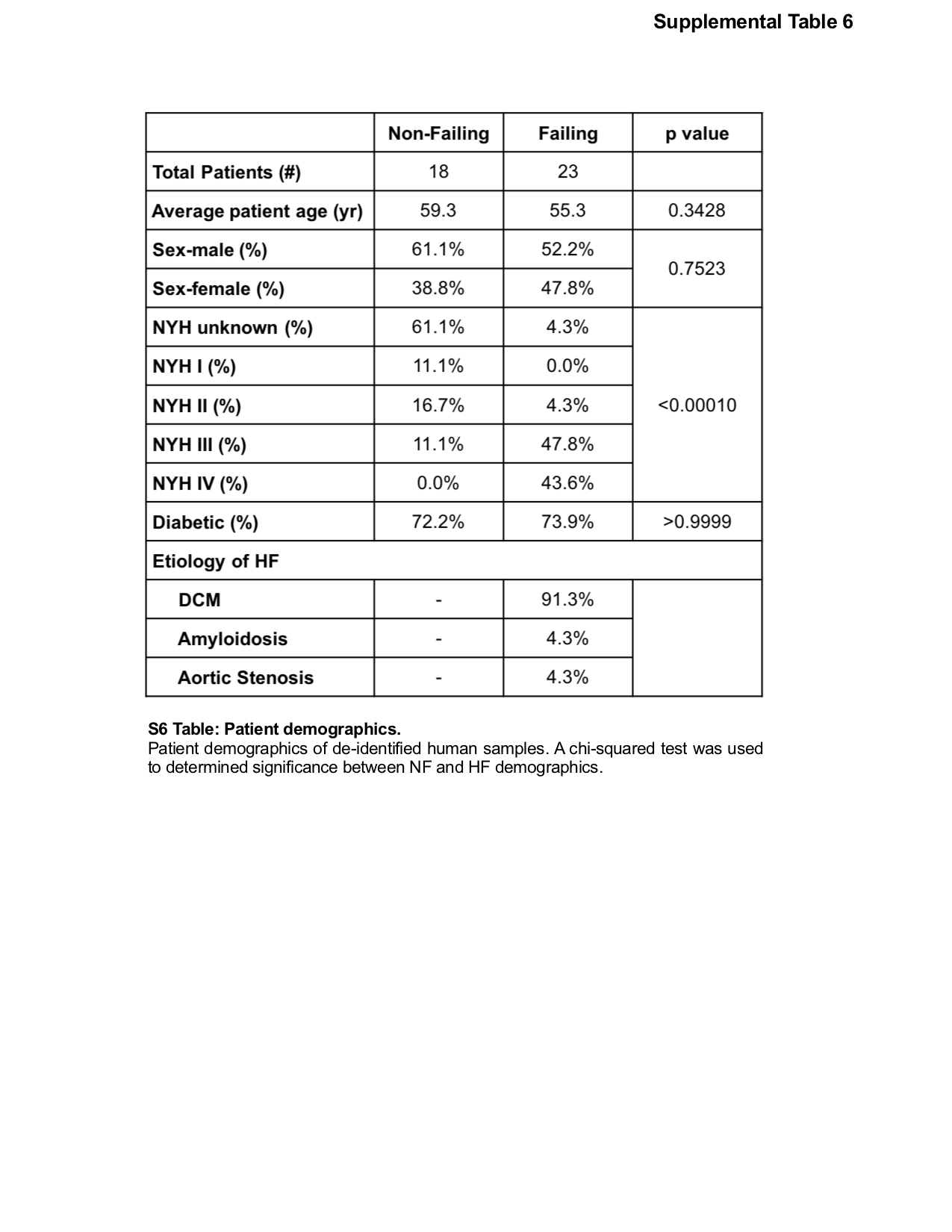

Supplement: S6 Table — Patient demographics of de-identified human samples. A chi-squared test was used to determined significance between NF and HF demographics. (TIFF) [file pone.0242250.s013.tiff]
