# Supplementary material for: Cardiomyocyte Oga haploinsufficiency increases O-GlcNAcylation but hastens ventricular dysfunction following myocardial infarction
Source: PLoS One. 2020 Nov 30;15(11):e0242250. doi: 10.1371/journal.pone.0242250 (PMC7703924; doi:10.1371/journal.pone.0242250)

Figure 1 (B and D) Western - OGA, O-GlcNAc

1B

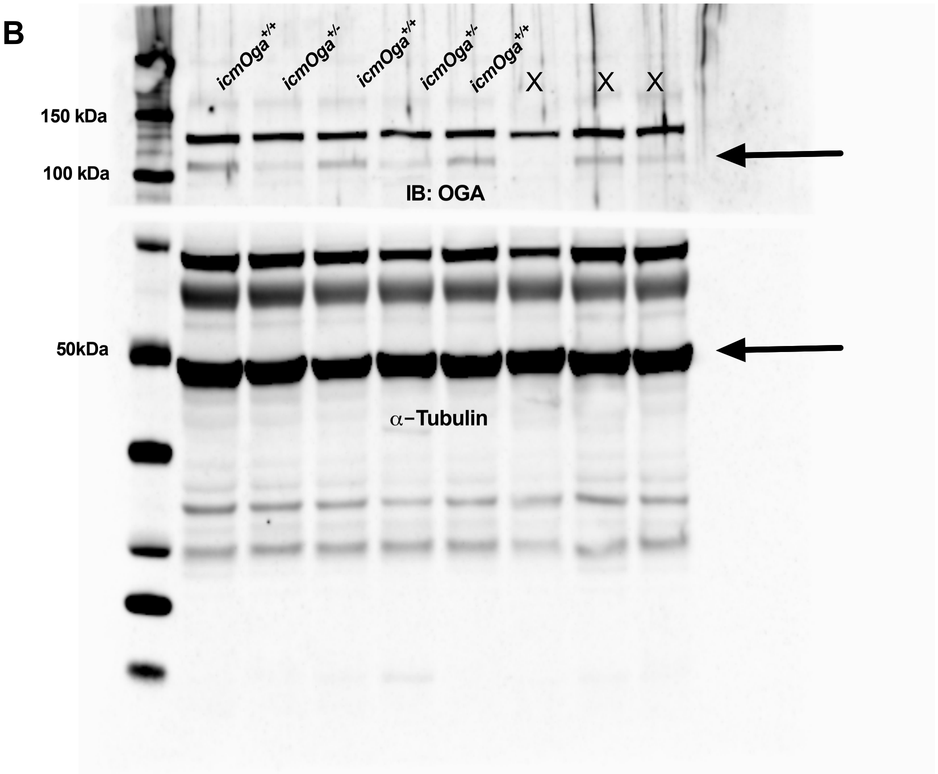

1D

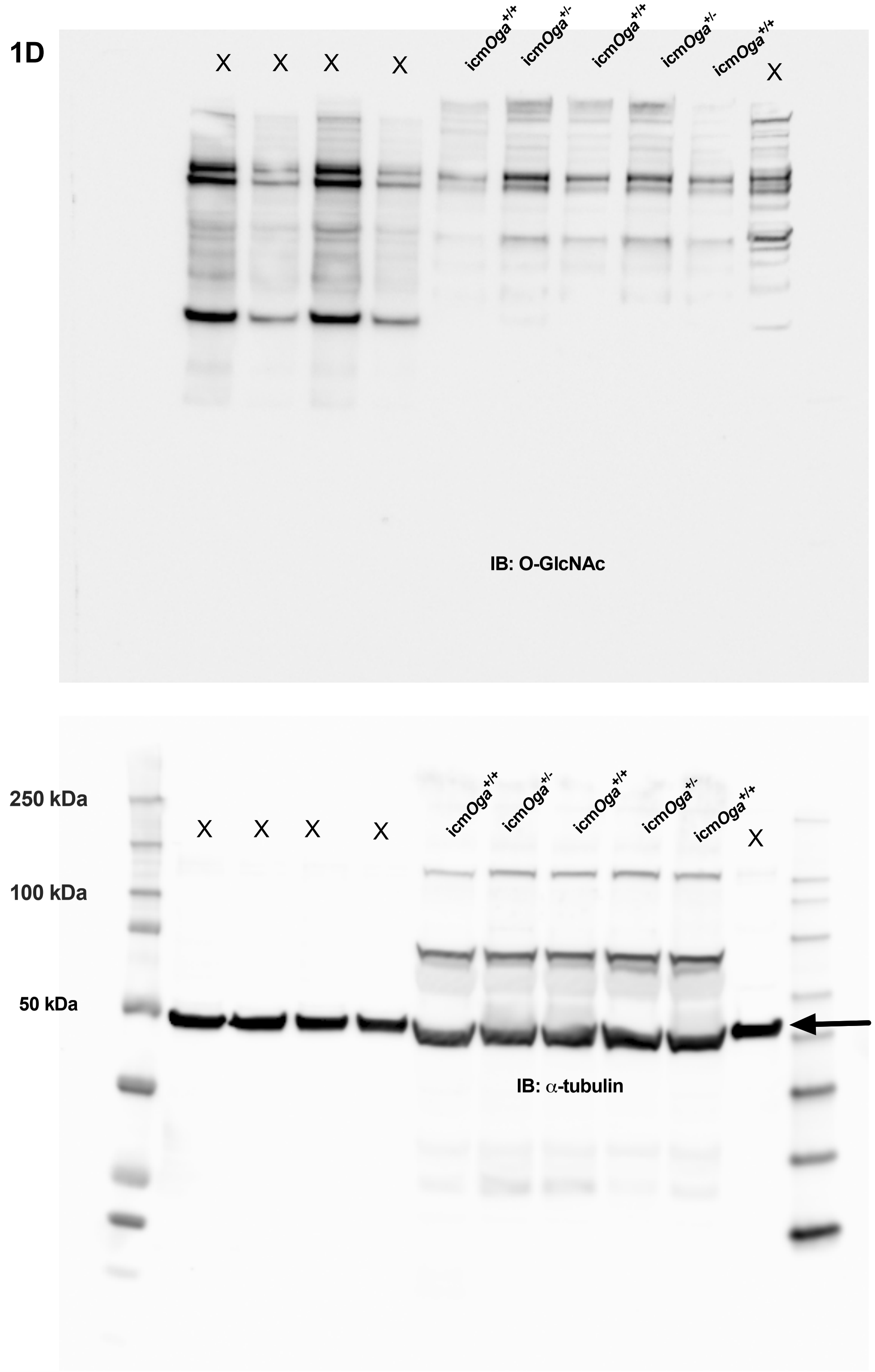

Fig 3 (A-C) Western - OGT, OGA, O-GlcNAc 1wk MI

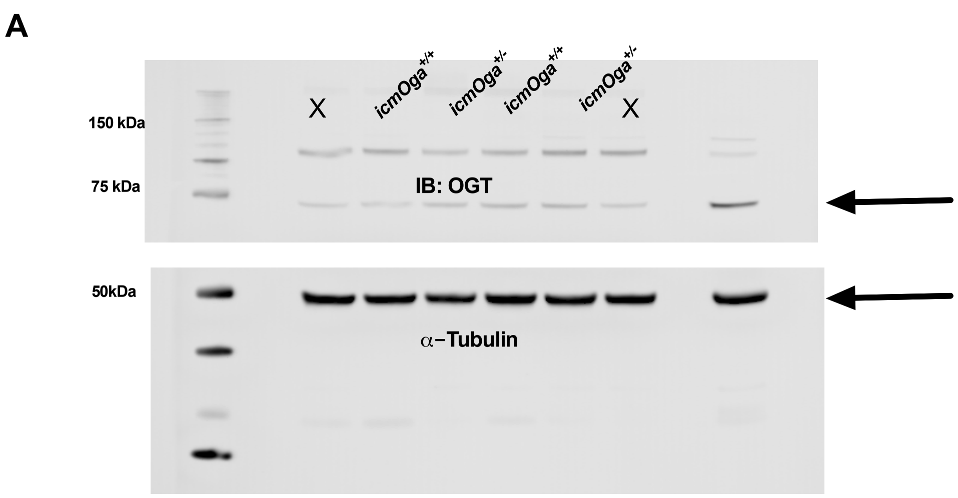

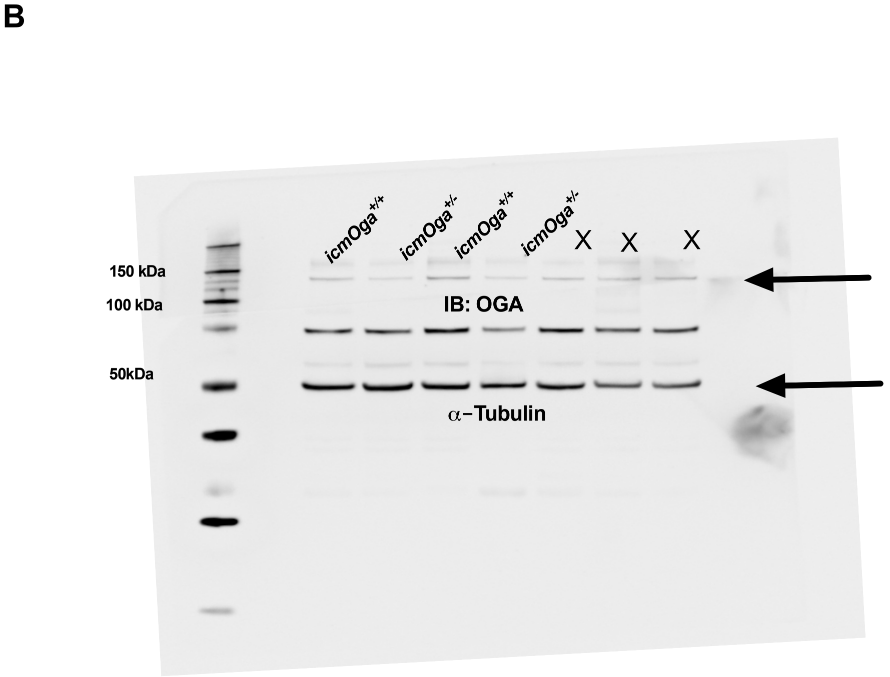

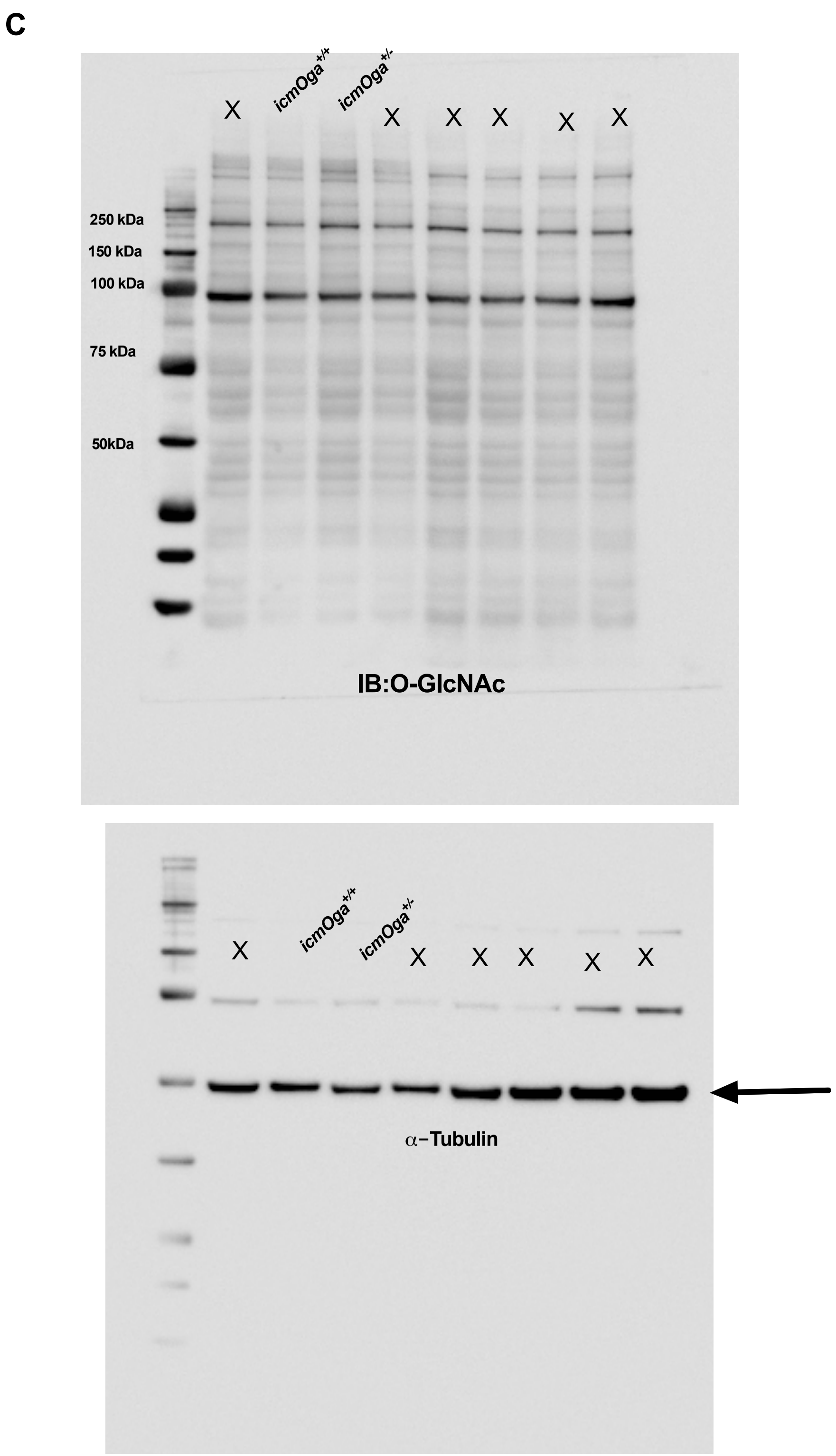

Fig 3 (D-F) Western - OGT, OGA, O-GlcNAc 4 wk MI

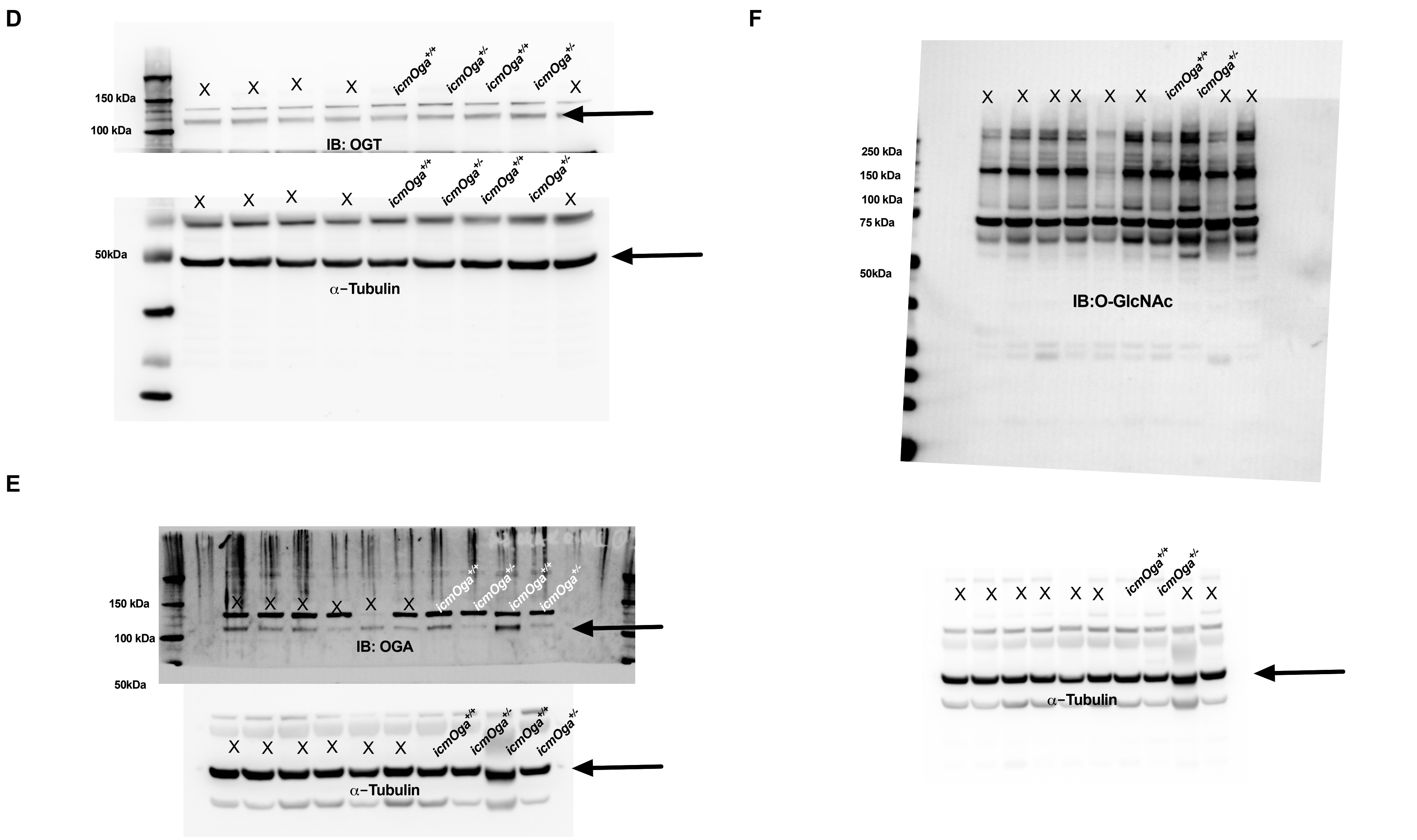

Figure 5B Western - OGA protein in Human HF

5B

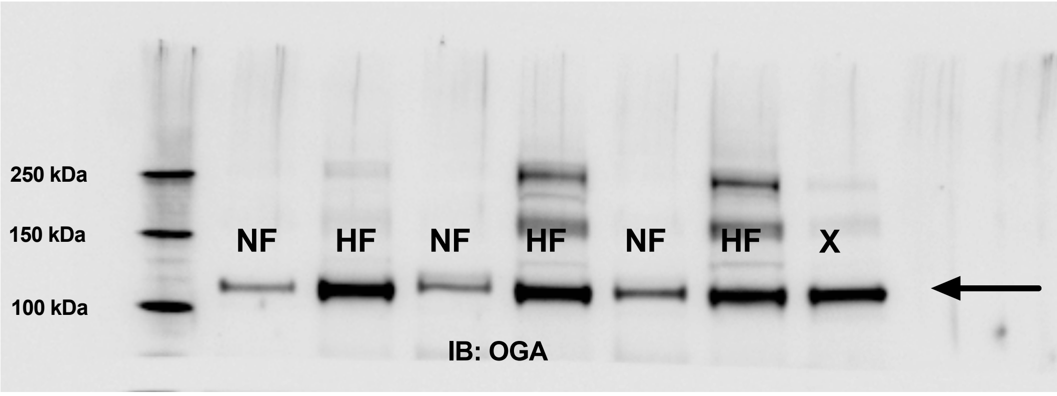

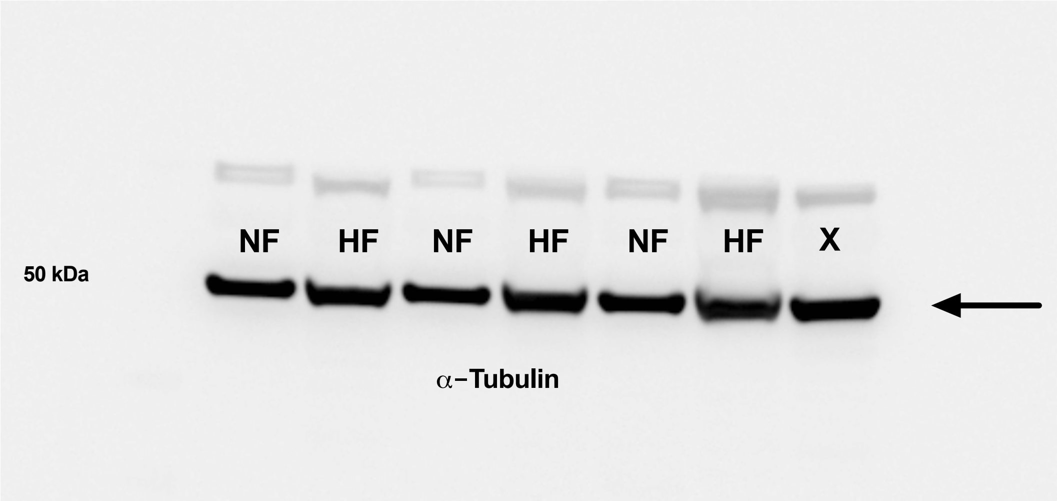

S2 Fig (A-C) Western - OGA

S2 A

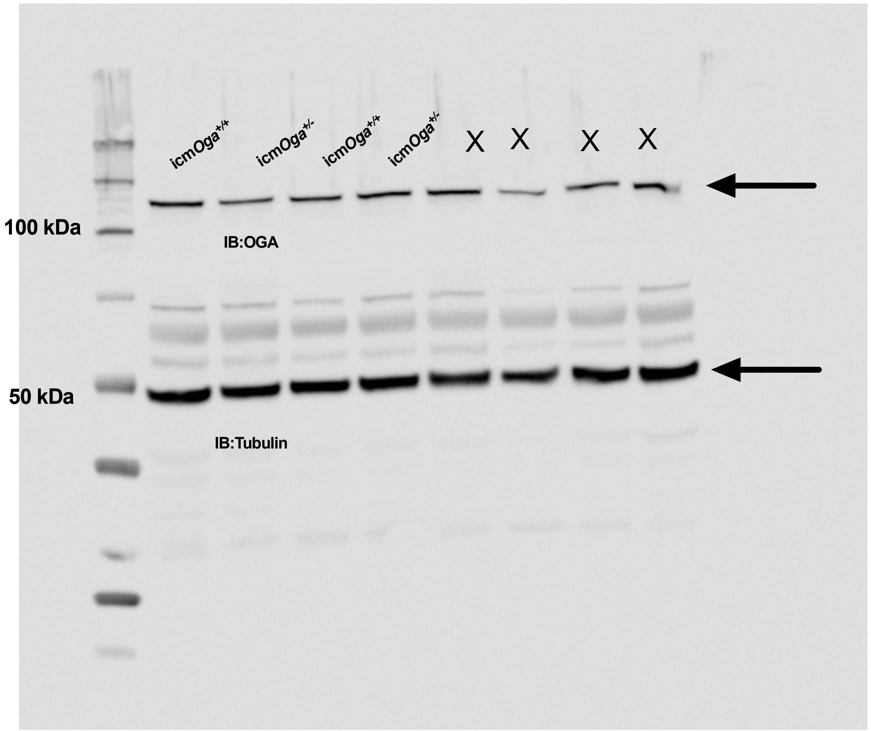

S2 B

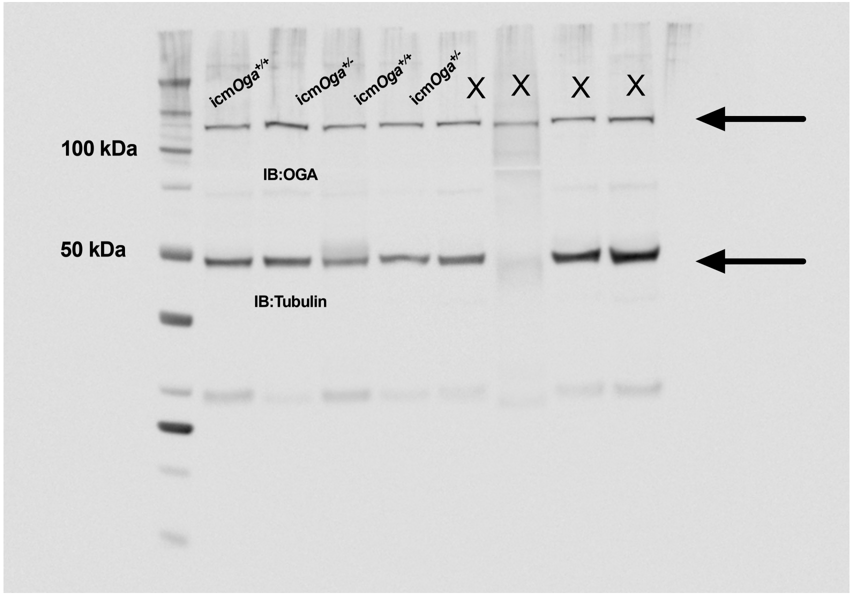

S2 C

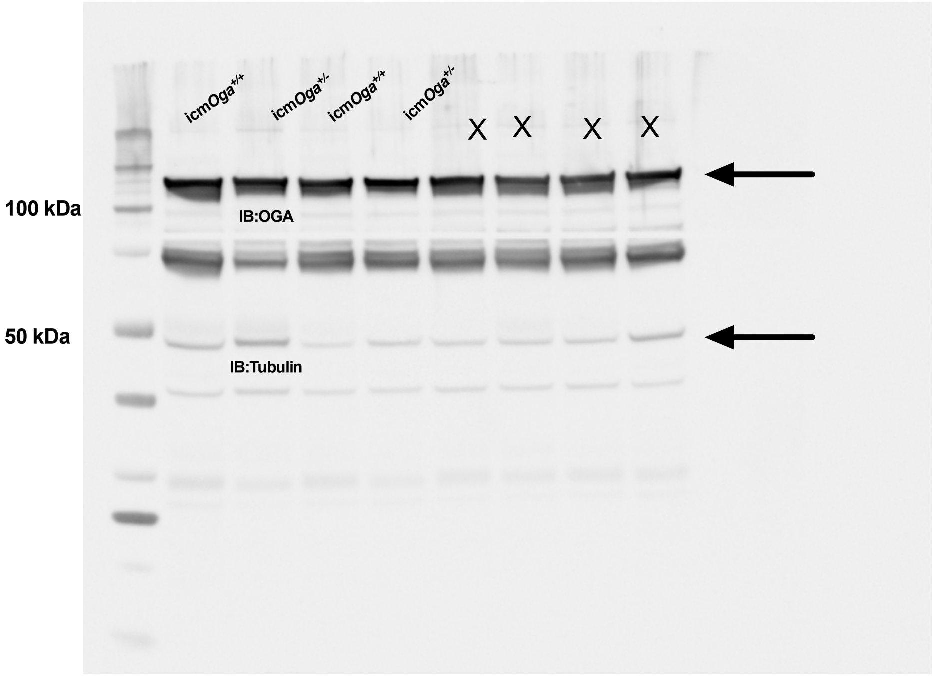

S7 Fig O-GlcNAc Western in Human HF

A

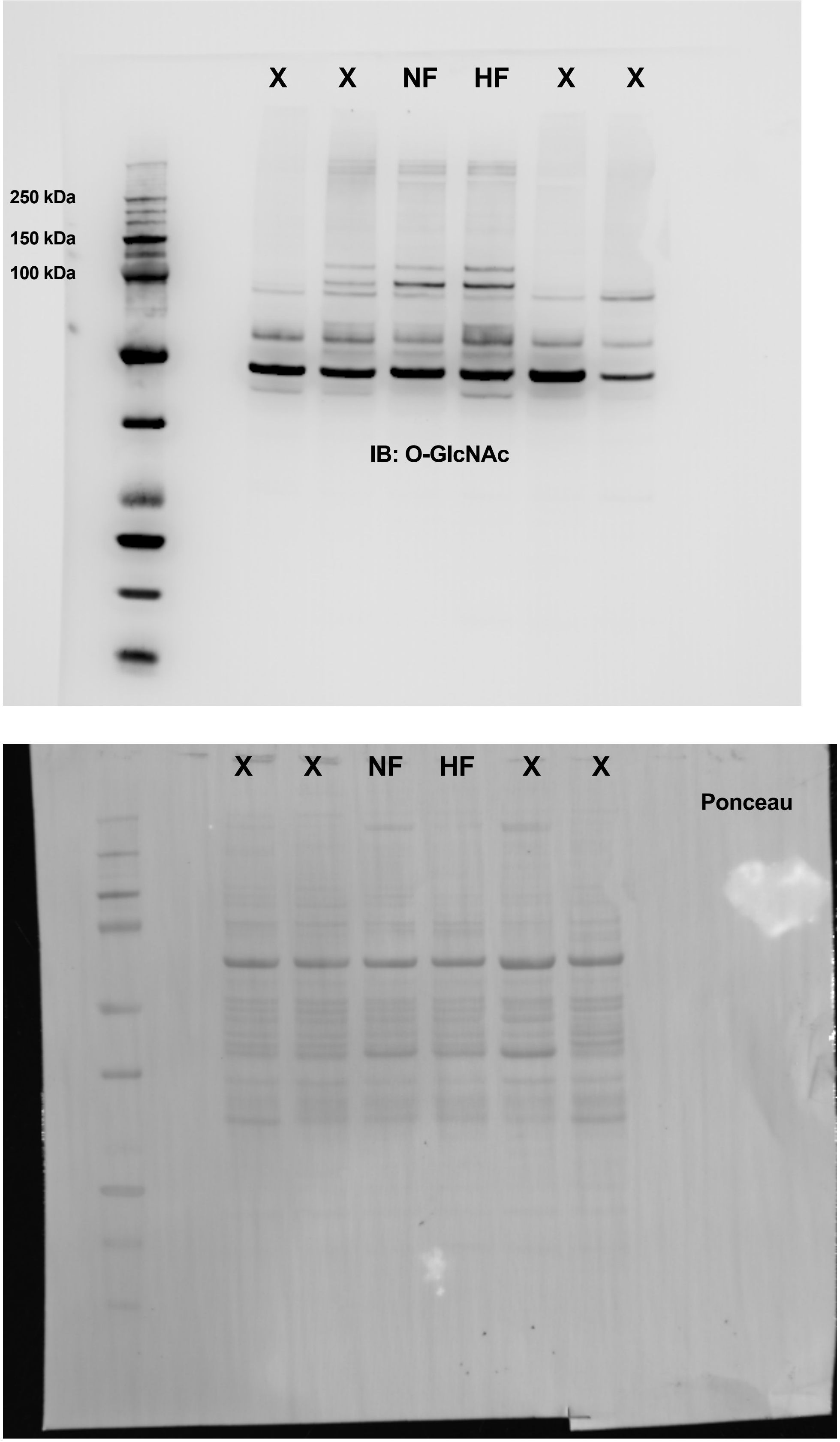

Supplement: S1 Raw images — (PDF) [file pone.0242250.s014.pdf]
